# Genetic gains in tropical maize hybrids across moisture regimes with multi-trait-based index selection

**DOI:** 10.3389/fpls.2023.1147424

**Published:** 2023-03-03

**Authors:** Ashok Singamsetti, Pervez H. Zaidi, Kaliyamoorthy Seetharam, Madhumal Thayil Vinayan, Tiago Olivoto, Anima Mahato, Kartik Madankar, Munnesh Kumar, Kumari Shikha

**Affiliations:** ^1^ Department of Genetics and Plant Breeding, Institute of Agricultural Sciences, Banaras Hindu University, Varanasi, India; ^2^ Asia Regional Maize Programme, The International Maize and Wheat Improvement Center (CIMMYT)-Hyderabad, Patancheru, India; ^3^ Department of Plant Science, Federal University of Santa Cataria, Florianópolis, Brazil; ^4^ Indian Council of Agricultural Research (ICAR) - Indian Agricultural Research Institute (IARI), Barhi, Jharkhand, India

**Keywords:** genotype-by-environment interaction (GEI), moisture regimes, multi-trait index, drought, waterlogging, climate-resilient maize, selection gains

## Abstract

Unpredictable weather vagaries in the Asian tropics often increase the risk of a series of abiotic stresses in maize-growing areas, hindering the efforts to reach the projected demands. Breeding climate-resilient maize hybrids with a cross-tolerance to drought and waterlogging is necessary yet challenging because of the presence of genotype-by-environment interaction (GEI) and the lack of an efficient multi-trait-based selection technique. The present study aimed at estimating the variance components, genetic parameters, inter-trait relations, and expected selection gains (SGs) across the soil moisture regimes through genotype selection obtained based on the novel multi-trait genotype–ideotype distance index (MGIDI) for a set of 75 tropical pre-released maize hybrids. Twelve traits including grain yield and other secondary characteristics for experimental maize hybrids were studied at two locations. Positive and negative SGs were estimated across moisture regimes, including drought, waterlogging, and optimal moisture conditions. Hybrid, moisture condition, and hybrid-by-moisture condition interaction effects were significant (*p* ≤ 0.001) for most of the traits studied. Eleven genotypes were selected in each moisture condition through MGIDI by assuming 15% selection intensity where two hybrids, viz., ZH161289 and ZH161303, were found to be common across all the moisture regimes, indicating their moisture stress resilience, a unique potential for broader adaptation in rainfed stress-vulnerable ecologies. The selected hybrids showed desired genetic gains such as positive gains for grain yield (almost 11% in optimal and drought; 22% in waterlogging) and negative gains in flowering traits. The view on strengths and weaknesses as depicted by the MGIDI assists the breeders to develop maize hybrids with desired traits, such as grain yield and other yield contributors under specific stress conditions. The MGIDI would be a robust and easy-to-handle multi-trait selection process under various test environments with minimal multicollinearity issues. It was found to be a powerful tool in developing better selection strategies and optimizing the breeding scheme, thus contributing to the development of climate-resilient maize hybrids.

## 1 Introduction

Maize yields in the Asian tropical rainfed environments are now becoming increasingly vulnerable to various climate-induced stresses, especially drought and waterlogging, which often come in combination to severely impact maize crops (Prasanna et al., 2021). The potential climate changes and abnormalities associated with a number of abiotic stresses severely affect the growth and development of crops (Brown and Funk, 2008; Lobell et al., 2008). Among many unpredictable changes, moisture stresses including low soil moisture stress (drought) and excess soil moisture stress (waterlogging/flooding) are the major constraints worldwide that result in almost 90% yield loss. A large portion of maize in the Asian tropics is cultivated in low-land tropics (<1,000 masl), representing a major mega-environment, followed by sub-tropical and tropical highlands (Zaidi et al., 2020). Approximately 80% of maize is being grown as a rainfed crop, prone to the vagaries of monsoon rains associated with a series of abiotic and biotic constraints. The erratic/uneven distribution of monsoon pattern leads to untimely rains, often causing intermittent dry spells/drought or excess soil moisture/waterlogging at different crop growth stages within the crop growing period (Zaidi et al., 2016a), and is a major factor responsible for the relatively low productivity in maize. The regular occurrence of temporary excessive soil moisture or waterlogging stress resulted in an almost 18% reduction in total maize in Southeast Asia (Zaidi et al., 2010). A few recent reports anticipated that the Asian tropics will experience a sharper increase in surface temperatures due to adverse weather conditions, resulting in shifting seasons and frequent drought and waterlogging at critical crop stages that could severely impact the maize production in the tropical regions (ADB, 2009; Lobell et al., 2011; Cairns et al., 2012; Zaidi et al., 2020).

In contrast, recent studies reported that the average global demand for maize has increased by 45% in 2020 when compared to 1997, with East Asia (85%) showing the highest increase in demand, followed by Sub-Saharan Africa (79%), Southeast Asia (70%), and South Asia (36%) (FAOSTAT, 2022; Ulfat et al., 2022). The development of improved germplasm with combined tolerance against extreme climate vagaries such as excess moisture and drought will be necessary in many areas of Asia, Africa, and Latin America (Zaidi et al., 2010). The international wheat and maize improvement center (CIMMYT)**–**Asia maize program largely focused on the development of breeding strategies to reach attainable yields across a range of environments by incorporating reasonable levels of cross-tolerance against a combination of major abiotic stresses without compromising grain yields under favorable/optimal growing conditions. It implies the maximum yield potential under optimal conditions along with a guarantee of average yields under stress-prone areas by improving the stress-resilient breeding pipelines through precision phenotyping, index-based selection for secondary traits, and stage-wise gateway screening (Zaidi et al., 2020). Under the various environmental conditions in India, developing climate-resilient and location-specific high-yielding hybrids became a primary goal of maize technologists. To achieve this, researchers must perform multi-environment trials (METs) prior to national trials. MET evaluates a set of genotypes under different test environments, which may be spatially varied (geographic locations), time-separated (seasons/years), different managed stresses (drought, waterlogging, low nitrogen, etc.), or a combination of any of these. METs can efficiently identify genotypes with a constant trait performance among different test environments (Yan and Kang, 2003). Comprehensive assessment of promising genotypes with higher yield potential and desirable agronomic characteristics is the key to breed hybrids with wide adaptability and stability. The undeniable existence of genotype-by-environment interaction (GEI) causes confusion in screening out promising genotypes, and it misleads the ranking pattern of test genotypes across environments. Thus, a deep understating of the degree and pattern of GEI across environments is crucial for successful crop improvement programs (Vaezi et al., 2019). Various analysis models and techniques such as analysis of variance (ANOVA), principal component analysis (PCA), cluster analysis, additive main effects and multiplicative interaction (AMMI), and genotype and genotype plus environment (GGE) biplots were developed to unravel the unpredictable effects of genotype, environment, and their interaction (Kendal, 2016).

Although grain yield is the most important characteristic of genotypes, other secondary traits are also common, such as days to flowering, plant height, ear height, ear length, and number of kernel rows (Gauch, 2013). The yield gains could be the result of the direct selection of grain yield that is accompanied by the desirable expression of other secondary traits. Previous reports on yield–trait relations provided a breakthrough for plant breeders in terms of defining a set of target traits to bring together new, high-performance hybrids (Nardino et al., 2016; Olivoto et al., 2017). It could be more efficient if the selection of genotypes is based on grain yield combined with other agronomic traits. However, an effective incorporation of multiple trait data without multicollinearity in the selection method has been a challenge for breeders. A widely used multi-trait selection index, the Smith–Hazel (SH) index (Smith, 1936; Hazel, 1943), is not being recommended for METs, where the presence of biased index coefficients and multicollinearity issues erodes the real genetic gains (Rocha et al., 2018; Olivoto et al., 2019a; Olivoto et al., 2019b; Jarquin et al., 2020; Woyann et al., 2020; Zuffo et al., 2020). Similarly, the recently proposed genotype × yield × trait (GYT) biplots (Yan and Frégeau-Reid, 2018) offer an effective and comprehensive analysis method for the evaluation of genotypes based on the combined grain yield and various evaluated agronomic traits. However, the method determines the influencing factors from yield–trait combinations by a classical linear multivariate model under diversified environments, but it does not involve any subjective weights and cutting points that provide a better picture of the strengths and weaknesses of test genotypes (Yan and Frégeau-Reid, 2018; Kendal, 2019; Nikolay et al., 2020; Singamsetti et al., 2022).

In that perspective, a novel multi-trait genotype–ideotype distance index (MGIDI) was proposed to select genotypes with desirable mean performances of multiple traits that overcome the fragility of classical linear indices (Olivoto and Nardino, 2021). A few previous attempts at multiple traits in the selection of maize hybrids with multi-environment data have been reported (Langner et al., 2019; Olivoto et al., 2021; Oliveira et al., 2020; Singamsetti et al., 2021; Peixoto et al., 2021; Shojaei et al., 2022; Yue et al., 2022c). The purpose of this research was mainly to select the promising maize hybrids based on multiple traits suitable for different moisture regimes including drought, waterlogging, and optimal conditions and across all moisture conditions. The study was carried out to reveal genetic parameters such as variance components, accuracy and heritability, inter-trait associations, and the patterns of GEI effects under individual and across moisture conditions.

## 2 Materials and methods

### 2.1 Plant material

The experimental material consists of 75 medium-duration maize hybrids including five commercial checks (**Supplementary Table 1**) leveraged from the Asian Regional Office, CIMMYT, Hyderabad, India. The 70 hybrids were developed from the biparental crosses obtained from a pool of 600 elite maize lines of the CIMMYT gene bank crossed with two abiotic stress-susceptible (mostly drought and waterlogging) testers, viz., CML451 and CL02450. These were globally released leading testers with high combining ability belonging to different heterotic groups, A and B, that resulted in test cross progeny with substantial stress tolerance. The materials were evaluated under different soil moisture stresses including drought, waterlogging, and optimal conditions by a stage-wise gateway process under the “Climate Resilient Maize for Asia” (CRMA) project supported by BMZ/GIZ, Germany. The studied hybrids were at the Stage III screening process that aimed to identify stress/location-specific hybrids and also those across different locations/stresses.

### 2.2 Testing environment

The trials across the different soil moisture conditions (described later in this article) were conducted at two locations, viz., Banaras Hindu University (BHU), Varanasi, India (Location 1) and CIMMYT, Hyderabad, ICRISAT Campus, India (Location 2), during winter 2017 and its subsequent summer–rainy season 2018. Location 1 is situated in the middle of the Indo-Gangetic plains of northern India, which experiences alternating floods and high temperatures in a year, while Location 2 is represented by the arid region of the Deccan Plateau with evident frequent occurrence of water scarcity followed by scattered rainfalls. The soil type of the experiment site at Location 1 was sandy loam, whereas it was shallow black soils at Location 2. The detailed information of seven test environments (E1 to E7) including three moisture conditions with season and location combinations is shown in **Table 1**. The meteorological data based on standard weeks including temperature and rainfall during the crop growing period at both locations are shown in **Supplementary Figure 1**.

**Table 1:** Details of test environments adopted for evaluation of 75 maize hybrids during the cropping seasons winter 2017–2018 and summer–rainy 2018.

Environment	Moisture regime	Crop season	Test location	Planting date
E1	Optimal	Winter	BHU, Varanasi	25 December 2017
E2	Managed drought	Winter	BHU, Varanasi	25 December 2017
E3	Managed drought	Winter	CIMMYT, Hyderabad	15 December 2017
E4	Optimal	Summer–rainy	BHU, Varanasi	6 July 2018
E5	Managed waterlogging	Summer–rainy	BHU, Varanasi	6 July 2018
E6	Optimal	Summer–rainy	CIMMYT, Hyderabad	8 July 2018
E7	Managed waterlogging	Summer–rainy	CIMMYT, Hyderabad	10 July 2018

BHU, Banaras Hindu University; CIMMYT, The International Maize and Wheat Improvement Center.

### 2.3 Experimental design and stress management

Test hybrids at each environment were sown in alpha lattice (0, 1) design (Patterson and Williams, 1976) with two replications. Manual sowing was done in two rows of 4 m length with a standard spacing of 75 cm between the rows and 20 cm within a row. A final plant population of 66,666 plants ha^−1^ was maintained by thinning in over-sown plots. All the recommended agronomic and cultural operations including irrigation were taken care of for optimal soil moisture (well-watered) trials. Drought and waterlogging phenotyping protocols by CIMMYT were strictly followed in both locations during managed stress trials (Zaman-Allah et al., 2016; Zaidi et al., 2016b). Field selection and protection measures were taken with utmost care to avoid other potential interruptions of plant growth and development during the moisture-stress period.

#### 2.3.1 Managed drought trials

Planting dates were adjusted to the dry period, i.e., winter or delayed *Rabi* season, and irrigation schedule was modified to impose drought stress at the reproductive stage of the crop. Cumulative growing degree days (GDD) were calculated from the day of life irrigation to ensure accurate stress intensity at the target stage of the crop at all the test environments. Withdrawal of irrigation at 550 cumulative GDD and release of stress by providing “rescue irrigation” at 1,000 cumulative GDD were followed to expose flowering to severe moisture stress (Zaman-Allah et al., 2016).


$$\text{Growing Degree Days }\left(\text{GDD}\right)=\sum^{}\left(\frac{T_{\text{max}}+T_{\text{min}}}{2}\right)-T_{\text{base}}$$

where *T*_max_ = maximum temperature, *T*_min_ = minimum temperature, and *T*_base_ = base temperature (10°C).

Progress in imposing stress was tracked by measuring moisture depletion at different soil depths up to 100 cm at 10-cm intervals with the “Delta-T PR2 soil moisture profile probe”. The probe was pre-installed in the fields where drought trials were conducted and weekly data were recorded from the first week after withdrawal of irrigation (a few days before anthesis), until the stress is relieved. Rescue irrigation was confirmed by the soil moisture content reaching the “permanent wilting point” (PWP), i.e., 16.8% v/v at the soil depth of 30–40 cm. Cumulative vapor–pressure deficit or ∑VPD values were also recorded as complementary to GDDs to endorse pausing and to resume the irrigation at 120 VPD and at 220 kPa VPD values, respectively.

#### 2.3.2 Managed waterlogging trials

Fields with proper irrigation and drainage facilities were selected for waterlogging phenotyping. These are well-leveled with no inclination, thus ensuring a depth of 10 ± 0.5 cm stagnation of water continuously for 7 days to impose waterlogging stress at the target crop stage, i.e., V_5_–V_6_ leaf stage or “knee-high” stage. Considering evaporation and seepage losses, additional need-based irrigation was provided to maintain the depth of water during the stress period. The crop was relieved from the stress by draining out the excess water in the experimental plots from the seventh day and a normal irrigation schedule was recommenced (Zaidi et al., 2016b).

### 2.4 Traits evaluated

During the flowering, maturity, and post-harvest stages of the crop, a total of 12 agronomic traits were recorded as per guidelines of standard abiotic stress phenotyping protocols by CIMMYT (Zaman-Allah et al., 2016; Zaidi et al., 2016b). The mean values at the plot level for the traits, viz., days to 50% anthesis (D50A), days to 50% silking (D50S), and anthesis–silking interval (ASI), were determined, whereas plant height (PH, in cm), ear height (EH, in cm), chlorophyll content (SPAD readings), ear length (EL, in cm), ear girth (EG, in cm), kernel number per row (KNR), kernel rows per ear (KRE), and test weight (TW, in g) were recorded as the mean of five randomly selected plants in each plot. SPAD readings were taken before and after imposing stress by the SPAD-502 plant chlorophyll meter. Moisture and shelling percent were measured by converting fresh weight of ears without husk per each plot to grain yield per hectare (GY, in t/ha) at 12.5% moisture (ASTM 2001).


$$\text{Grain Yield }\left(\text{t}/\text{ha}\right)=\frac{\text{Fresh ear weight }\left(\text{kg}/\text{plot}\right)\times 10\times \left(100-\text{MC}\right)\times \text{SH}}{\left(100-\text{Adjusted MC}\right)\times \text{Plot area }\left({\text{m}}^{2}\right)}$$

where MC is moisture content, SH is shelling percentage, and adjusted MC is the required standardized moisture percentage (12.5%)

### 2.5 Data analysis and software

The combined data from all the test environments were checked using the Shapiro–Wilk test (Shapiro and Wilk, 1965) for ANOVA residuals and confirmed normal distribution. Homogeneity of the data of an individual environment as well as of similar moisture conditions was confirmed through Bartlett’s test (Bartlett, 1937).

#### 2.5.1 Variance component analysis

For each soil moisture condition, the traits were initially fitted into a linear mixed-effect model by considering genotype, genotype-by-environment, and incomplete blocks within complete replicates as random effect and locations and complete replicates as fixed effect (Olivoto et al., 2019a). The following standard linear mixed model (Yang, 2007) was computed with the function *gamem_met()* from the metan package (Olivoto and Lúcio, 2020).


y=Xβ+Zu+ϵ

where **y** is a vector of response variable (such as grain yield), **β** is a vector of fixed effects, **u** is a vector of random effects; **X** is a design matrix of 0s and 1s relating **y** to **β**, **Z** is a design matrix of 0s and 1s relating **y** to **u**, and **ε** is a vector of random errors.

The estimates of variance components were obtained by REstricted Maximum Likelihood (REML) using the expectation-maximum algorithm (Dempster et al., 1977). A likelihood ratio test (LRT) with a two-tailed chi-square test with one degree of freedom was performed to test the significance of the random effects. Broad-sense heritability based on genotypic mean performance ( $h_{mg}^{2}$ ) was estimated as follows:


$$h_{mg}^{2}=\frac{{\hat{\sigma }}_{g}^{2}}{\left[{\hat{\sigma }}_{g}^{2}+\frac{{\hat{\sigma }}_{i}^{2}}{e}+\frac{{\hat{\sigma }}_{e}^{2}}{eb}\right]}$$

Where ${\hat{\sigma }}_{g}^{2}$ , ${\hat{\sigma }}_{i}^{2},\;$ and ${\hat{\sigma }}_{e}^{2}$ are the variances related to genotypes, genotype–environment interaction, and error terms, respectively; *e* and *b* are the number of environments and blocks per environments, respectively.

#### 2.5.2 Genetic correlations

To better understand the inheritable relationships among traits and to see if these relationships are changed across moisture regimes, a genetic correlation was performed in each moisture condition. The correlation matrix was represented as network plots.

#### 2.5.3 The multi-trait genotype–ideotype distance index

The estimation of MGIDI values for test hybrids in each moisture condition was based on two-way Best Linear Unbiased Predictions (BLUPs) for each genotype (row) and trait (column) and was carried out in four steps, i.e., rescaling of the studied traits, exploratory factor analysis (EFA) to reduce the dimensionality, planning for ideotype with maximum rescaled value, and calculation of Euclidean distance between the genotypes and ideotype planned as the MGIDI index (Olivoto and Nardio, 2021). Rescaling the traits was performed so that all have a similar range, i.e., 0–100. The rescaled value (*r*X*_ij_*) of the *j*th trait (column) of the *i*th genotype (row) was calculated using the following formula:


$$rX_{ij}=\frac{\eta_{nj}-\varphi_{nj}}{\eta_{0j}-\varphi_{0j}}\times \left(\theta_{ij}-\eta_{oj}\right)+\eta_{nj}$$

where *η*_0*j*_ and *φ*_0*j*_ are the original maximum and minimum values for the trait *j*, respectively; *θ*_*ij*_ is the original value for the *j*th trait of the *i*th genotype/hybrid; and *η*_*nj*_ and *φ*_*nj*_ are the new maximum and minimum values for the trait *j* after rescaling, respectively. The values for *η*_*nj*_ and *φ*_*nj*_  are chosen according to the desirability as follows. For the traits PH, EH, SPAD, EL, EB, TW, KRE, KNR, and GY in which positive gains are desired, we used *η*_*nj*_  = 100 and *φ*_*nj*_ = 0. For D50A, A50S, and ASI in which negative gains are desired, we considered *η*_*nj*_ = 0 and *φ*_*nj*_ = 100. The correlation matrix of the original set of trait values (X*_ij_*) was maintained by the rescaled trait values (*r*X*_ij_*) in a two-way table in which each column with a range of 0–100 made a selection, i.e., increase or decrease.

In the second step, the factorial scores of each test hybrid/genotype was estimated by performing EFA with rescaled values (*r*X*_ij_*) to group correlated traits into “*factors*”. By assuming *p* and *f* are the number of traits included and common factors retained through EFA, respectively, the scores were calculated as follows:


X=μ+Lf+ϵ

where ***X*** is a *p* × 1 vector of rescaled observations; ***µ*** is a *p* × 1 vector of standardized means; ***L*** is a *p* × *f* matrix of factorial loadings; ***f*** is a *p* × 1 vector of common factors; and ε is a *p* × 1 vector of residuals. Furthermore, the initial loadings were obtained by the traits having more than one eigenvalue that are acquired from the correlation matrix of *r*X*ij*. Then, final loadings were estimated by using *varimax* rotation criterion (Kaiser, 1958; Olivoto et al., 2019a) as given by:


$$F=Z{\left(A^{T}R^{-1}\right)}^{T}$$

where ***F*** is a *g* × *f* matrix with the factorial scores; ***Z*** is a *g × p* matrix with the standardized means (rescaled); ***A*** is a *p* × *f* matrix of canonical loadings; and ***R*** is a *p* × *p* correlation matrix between the traits. *g*, *f*, and *p* denote the number of test hybrids/genotypes (rows), factors retained (FA), and traits analyzed, respectively.

The ideotype (ID) was designed by assuming that it has the highest rescaled value, i.e., 100 for all the traits analyzed. Thus, the ID can be defined by 1 × *p* vector ID such that ID = [100, 100, …., 100]. The final scores for ID were also obtained according to the above formula. Finally, the MGIDI values were computed with the function *mgidi()* from the metan package. If *g* and *f* are the number of genotypes/rows and factors retained, respectively, the MGIDI for the *i*th genotype (MGIDI*_i_*) is calculated as follows:


$$MGIDI_{i}={\left[\sum_{j=1}^{f}{\left(\gamma_{ij}-\gamma_{j}\right)}^{2}\right]}^{0.5}$$

where *γ*_*ij*_  is the score of the *i*th genotype (row) in the *j*th factor (*i* = 1, 2, …, *gf*) and *γ*_*j*_  is the *j*th score of the ideotype. The genotypes with the lowest MGIDI values, i.e., genotypes closer to the ID, exhibited the desired values for all the traits studied. The strengths and weaknesses of a genotype were represented by the proportion of the MGIDI index of the *i*th row/genotype explained by the *j*th factor (*ij*) estimated as follows:


$$\omega_{ij}=\frac{\sqrt{D_{ij}^{2}}}{\sum_{j=1}^{f}\sqrt{D_{ij}^{2}}}$$

where *D_ij_* is the distance between the *i*th genotype (row) and the ID for the *j*th factor. Low contributions of a factor specify that the traits within that factor are similar to the ideotype designed.

#### 2.5.4 Selection differential

The hybrids were selected under different soil moisture regimes through MGIDI values by assuming a selection intensity of ~15% and the selection differential in the percentage of population mean (*ΔS%* ) was then computed for each trait as follows:


$$\Delta S\%=\frac{\left(X_{S}-X_{0}\right)}{X_{0}}\times 100$$

where *X_s_* and *X*_0_ are the mean performance value of the selected hybrids and population (original population) mean, respectively.

### 2.6 Statistical software

All the statistical analyses were carried out on the RStudio, R version 4.1.2 (R Core Team, 2021) software with “metan” version v1.15.0 (Olivoto and Lúcio, 2020) and “ggplot2” version 3.3.4 (Wickham, 2016) packages. Functions such as *gamem_met()* for genotype analysis in multi-environments using mixed-effect or random-effect models, *gmd()* for extracting variance components, and *mgidi()* for the computation of MGIDI values were supplied. The network plots of the pairwise correlation data frame were constructed by using “corrr” package version 0.4.4 (Kuhn et al., 2022).

## 3 Results

### 3.1 Mean performances

**Figure 1:**
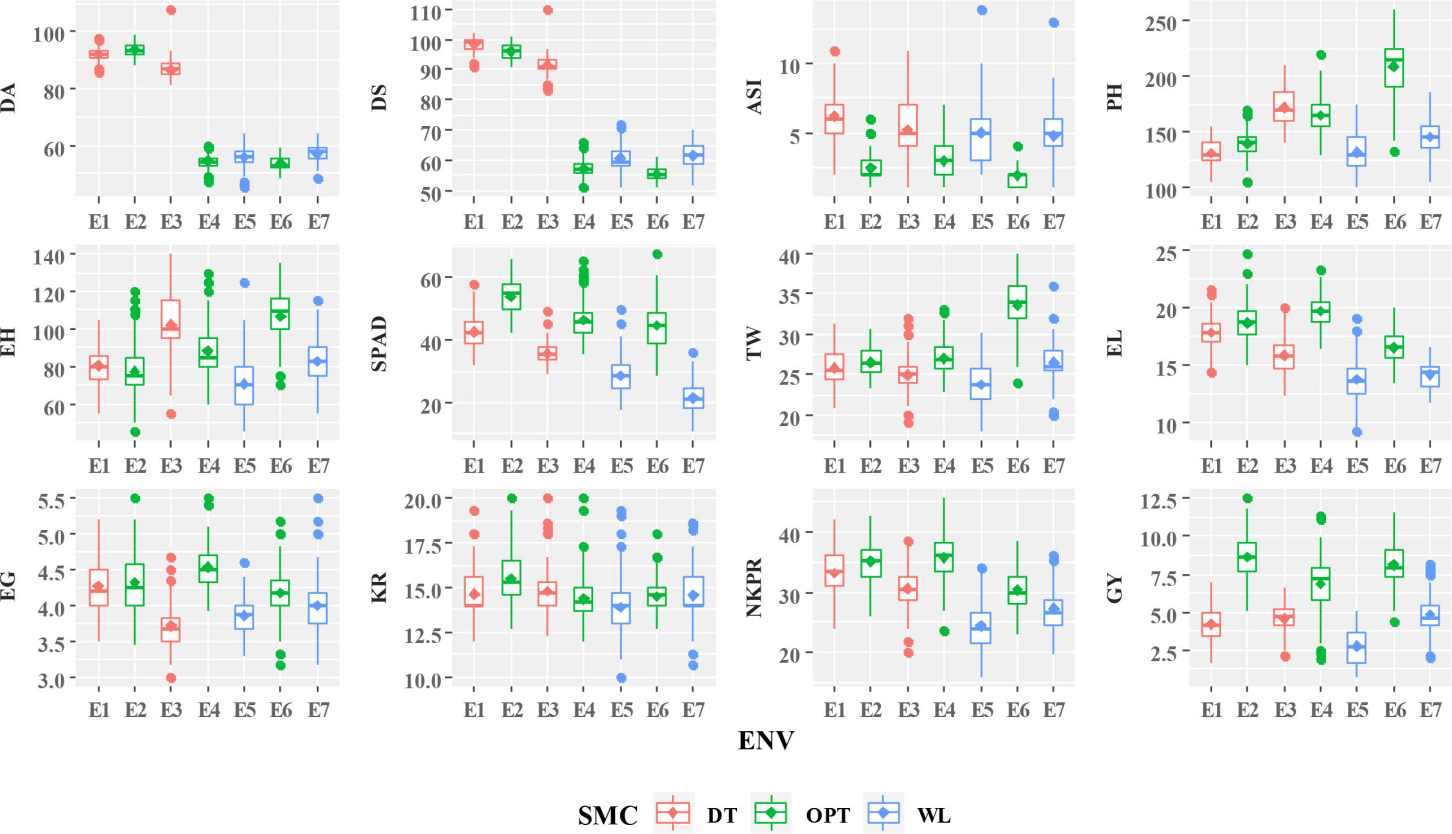
Mean performances of studied traits of 75 maize genotypes under different soil moisture conditions including drought (DT), optimal (OPT), and waterlogging (WL). DA, days to 50% anthesis (#); DS, days to 50% silking (#); ASI, anthesis–silking interval (#); PH, plant height (cm); EH, ear height (cm); SPAD, chlorophyll content (SPAD readings); TW, test weight (g); EL, ear length (cm); EG, ear girth (cm); KR, number of kernel rows per ear (#); NKPR, number of kernels per row (#); GY, grain yield (t/ha); SMC, soil moisture condition; DT, drought; WL, waterlogging; OPT, optimal; ENV, environments.

Mean performances of 75 genotypes for 12 agronomic traits over seven test environments including three moisture regimes are presented in **Figure 1**. Hybrids 22 (ZH161289; 10.48 t/ha) followed by 30 (ZH161047, 10.35 t/ha), 6 (ZH161361; 9.87 t/ha), 14 (ZH161303; 9.85 t/ha), and 19 (ZH161458; 9.77 t/ha) were identified as top yielders under optimal soil moisture; hybrids 36 (ZH161053; 6.07 t/ha) followed by 22 (ZH161289; 5.96 t/ha), 9 (ZH161384; 5.89 t/ha), 44 (ZH161063; 5.89 t/ha), and 41 (ZH161051; 5.81 t/ha) were the top yielders under drought; and hybrids 30 (ZH161047; 6.21 t/ha) followed by 49 (ZH161398; 6.06 t/ha), 22 (ZH161289; 5.94 t/ha), 14 (ZH161303; 5.90 t/ha), and 44 (ZH161063; 5.57 t/ha) were the top yielders under waterlogging (**Supplementary Tables 2A–C**). The hybrids ZH161053, ZH161289, ZH161063, ZH161398, and ZH161047 were found to be common under all three moisture conditions in terms of grain yield.

### 3.2 Variance components across moisture regimes

**Figure 2:**
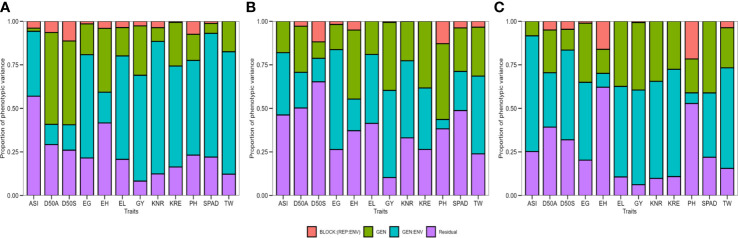
The proportion of phenotypic variance for 12 agronomic traits of 75 maize hybrids evaluated under **(A)** optimal, **(B)** drought, and **(C)** waterlogging during winter 2017 and summer–rainy 2018. D50A, days to 50% anthesis; D50S, days to 50% silking; ASI, anthesis–silking interval; PH, plant height; EH, ear height; SPAD, chlorophyll content; EL, ear length; EG, ear girth; KRE, number of kernel rows per ear; KNR, number of kernels per a row; TW, test weight and GY, grain yield.

The genotype had a highly significant effect (*p* ≤ 0.001, *p* ≤ 0.01, and *p* ≤ 0.05) for most of the studied traits except for ASI, SPAD, and KNR under optimal moisture conditions, D50S and EG under drought, and D50 and ASI under waterlogging according to LRT (**Table 2**). The test showed that the GEI revealed a highly significant effect (*p* ≤ 0.001 and *p* ≤ 0.01) on all the studied traits except PH across three moisture conditions, D50S under drought, and EH under waterlogging. Similarly, the environment effect was highly significant (*p* ≤ 0.001 and *p* ≤ 0.01) for all the evaluated traits except for KRE under optimal and drought environment and D50S, ASI, and EL under waterlogging stress conditions. The proportions of total variation explained by genotype, environment, and their interactions (GEI) under individual moisture regimes are shown in **Figure 2** (**Supplementary Table 3**). The accuracy of hybrid selection for the studied traits ranged from 0.40 (PH) to 0.96 (D50A and D50S) under optimal conditions, 0.31 (SPAD) to 0.88 (PH) under drought, and 0.65 (ASI) to 0.93 (SPAD) under waterlogging conditions. The coefficients of determination for GEI effects ( $R_{ge}^{2}$ ) were high for GY, KNR, KRE, TW, EG, and EL under all the moisture conditions, indicating that GEI holds an important part of the phenotypic variance component. Most of the traits showed high heritability on genetic mean basis ( $h_{mg}^{2}$ > 0.60) under optimal (except ASI and SPAD) and waterlogging conditions (except D50S and ASI), whereas only a few traits such as PH, EH, KRE, and GY showed high heritability.

**Table 2:** Likelihood ratio test and genetic parameters for 12 agronomic traits of 75 maize hybrids under three moisture regimes, namely, optimal, drought, and waterlogging.

Trait	Genetic Parameters									
	LRT_g_	LRT_ge_	$\sigma_{p}^{2}$	$R_{ge}^{2}$	$h_{mg}^{2}$	As	E/F	CV_g_	CV_r_	CV_g_/CV_r_
Optimal										
D50A	95.49^***^	12.33^***^	4.60	0.12	0.93	0.96	11,807.00^***^	2.32	1.72	1.34
D50S	83.10^***^	19.17^***^	5.80	0.16	0.92	0.96	7,061.00^***^	2.40	1.77	1.36
ASI	0.15^ns^	29.43^***^	1.09	0.39	0.16	0.40	14.55^***^	5.79	32.47	0.17
PH	6.60*	112.39^ns^	297.50	0.59	0.61	0.78	221.3^***^	3.91	4.87	0.81
EH	45.10^***^	14.14^***^	169.0	0.18	0.86	0.93	81.64^***^	8.68	9.21	0.94
SPAD	0.87ns	167.65^**^	33.57	0.72	0.33	0.57	58.29^***^	2.86	5.63	0.50
EL	7.06^***^	144.21^***^	2.03	0.62	0.62	0.78	90.38^***^	3.15	3.56	0.89
EG	8.14^**^	145.25^***^	0.12	0.60	0.63	0.79	27.43^***^	3.33	3.67	0.91
KRE	14.94^***^	175.24^***^	1.39	0.58	0.73	0.85	20.66^ns^	4.00	3.23	1.24
KNR	1.57^ns^	239.05^***^	12.67	0.79	0.40	0.63	48.17^***^	2.95	3.71	0.79
TW	7.09^**^	258.82^***^	5.89	0.70	0.61	0.78	235.2^***^	3.49	2.92	1.19
GY	18.19^***^	274.26^***^	2.08	0.62	0.75	0.86	31.48^***^	9.76	5.27	184
Managed Drought Stress										
D50A	10.31^**^	8.71^**^	7.10	0.21	0.54	0.73	201.9^***^	1.54	2.11	0.72
D50S	1.89 ^ns^	2.52 ^ns^	5.12	0.15	0.29	0.53	245.8^***^	0.73	1.92	0.38
ASI	4.18^*^	23.14^***^	3.87	0.35	0.38	0.62	14.99^***^	14.56	23.31	0.62
PH	34.01^***^	1.12 ^ns^	195.3	0.06	0.78	0.88	288.3^***^	6.09	5.71	1.06
EH	21.91^***^	10.65^**^	186.2	0.19	0.68	0.83	124.2^***^	9.41	9.11	1.03
SPAD	9.10^***^	9.47^***^	19.16	0.23	0.51	0.31	99.31^***^	5.59	7.81	0.71
EL	4.39*	30.36^***^	2.04	0.39	0.38	0.62	75.96^***^	3.71	5.48	0.68
EG	2.11 ^ns^	67.28^***^	0.11	0.58	0.30	0.53	96.11^***^	3.17	4.29	0.74
KRE	16.02^***^	45.13^***^	1.68	0.35	0.61	0.78	0.83^ns^	5.45	4.53	1.20
KNR	5.61^*^	44.53^***^	11.42	0.44	0.42	0.65	37.04^***^	5.04	6.11	0.83
TW	8.26^**^	57.25^***^	5.05	0.46	0.49	0.70	18.11^***^	4.96	4.33	1.09
GY	13.85^***^	138.34^***^	0.96	0.50	0.59	0.76	12.19^***^	13.96	7.17	1.95
Managed Waterlogging Stress										
D50A	7.80**	20.1^***^	9.15	0.33	0.77	0.88	4.07^*^	2.66	3.36	0.79
D50S	1.61 ^ns^	47.31^***^	13.33	0.54	0.55	0.74	1.27^ns^	2.06	3.37	0.61
ASI	0.68^ns^	89.31^***^	4.77	0.66	0.43	0.65	0.73^ns^	12.93	22.46	0.57
PH	9.08^**^	0.76^ns^	233.0	0.08	0.81	0.89	4.04^*^	4.86	8.01	0.61
EH	4.33^*^	1.00^ns^	148.7	0.09	0.71	0.84	9.70^***^	5.92	12.56	0.47
SPAD	17.76^***^	57.76^***^	26.73	0.37	0.86	0.93	139.4^***^	13.24	9.67	1.37
EL	12.55^***^	144.24^***^	2.38	0.52	0.82	0.91	1.51^ns^	6.78	3.61	1.88
EG	11.58^***^	72.56^***^	0.11	0.45	0.81	0.90	6.34^**^	4.87	3.77	1.29
KRE	6.57^*^	161.57^***^	2.20	0.62	0.74	0.86	10.01^**^	5.48	3.44	1.60
KNR	10.42^**^	161.26^***^	13.06	0.56	0.78	0.89	33.33^***^	8.21	4.38	1.88
TW	5.05^*^	105.18^***^	6.34	0.59	0.71	0.84	49.63^***^	4.81	3.95	1.22
GY	12.99^***^	196.68^***^	1.45	0.55	0.83	0.91	157.3^***^	19.74	7.88	2.51

***significant at 0.1% (p< 0.001); **significant at 1% (p< 0.01); *significant at 5% (p< 0.05); ns, nonsignificant. LRT_g_ and LRT_ge_, Likelihood ratio tests for genotype and genotype-by-environment interaction (GEI), respectively; $\sigma_{p}^{2}$ , phenotypic variance; $R_{ge}^{2}$ , the coefficient of determination for GEI effects; $h_{mg}^{2}$ mg, heritability of the genotypic mean; As, the accuracy of genotype selection; E/F, the F value for environment effects; CV_g_ and CV_r_, the genotypic and variation coefficients of variation, respectively. D50A, days to 50% anthesis; D50S, days to 50% silking; ASI, anthesis–silking interval; PH, plant height; EH, ear height; SPAD, chlorophyll content; EL, ear length; EG, ear girth; KRE, number of kernel rows per ear; KNR, number of kernels per a row; TW, test weight and GY, grain yield.

### 3.3 Loadings and factor description for MGIDI

According to final loadings obtained from PCA followed by EFA, three factors (FAs with more than 1 eigenvalue) contributing 68.7% of total variability were retained under optimal conditions whereas four factors with 69.6% and four factors with 76.4% towards total variability were retained under drought and waterlogging conditions, respectively (**Table 3**). Under optimal conditions, PH, EH, SPAD, EL, KNR, and GY belonged to FA1; D50A, D50S, ASI, and TW were included in FA2; and EG and KRE were included in FA3. Among four factors retained in drought, FA1 included TW, EL, KNR, and GY; FA2 included D50A, D50S, and ASI; FA3 included EG and KRE; and FA4 included PH, EH, and SPAD. Similarly, under waterlogging, FA1 included SPAD, TW, EL, KNR, and GY; FA2 included D50A, PH, and EH; FA3 included EG and KRE; and F4 included D50S and ASI.

**Table 3:** Eigenvalues, explained variance, cumulative variance, and final loadings of factors retained after superposition by exploratory factor analysis.

Traits	Optimal			Managed Drought				Managed Waterlogging			
	FA1	FA2	FA3	FA1	FA2	FA3	FA4	FA1	FA2	FA3	FA4
D50A	0.14	−0.88	0.11	0.11	0.98	−0.04	0.15	−0.30	0.67	−0.19	0.38
D50S	0.07	−0.95	−0.03	−0.34	0.78	−0.10	−0.17	−0.32	0.38	−0.13	0.83
ASI	−0.23	−0.52	−0.44	−0.48	−0.59	−0.06	−0.38	−0.10	−0.33	0.07	0.82
PH	−0.82	0.16	0.20	−0.23	−0.19	0.12	−0.81	−0.12	−0.83	−0.14	0.05
EH	−0.76	0.35	0.10	−0.07	0.02	−0.02	−0.92	−0.17	−0.77	−0.18	0.14
SPAD	−0.59	−0.20	0.45	−0.34	0.07	−0.17	−0.36	−0.65	−0.19	−0.48	0.21
TW	−0.23	−0.52	0.33	−0.44	0.25	0.44	0.31	−0.77	0.25	−0.04	0.19
EL	−0.71	−0.22	0.05	−0.79	−0.03	0.12	−0.06	−0.86	−0.14	0.15	−0.03
EG	−0.22	−0.06	0.78	−0.18	0.09	−0.73	0.10	0.01	−0.22	−0.83	0.15
KRE	−0.31	−0.01	0.82	−0.08	0.03	−0.81	−0.04	−0.16	0.03	−0.87	−0.13
KNR	−0.83	−0.04	0.24	−0.74	0.01	−0.31	−0.28	−0.67	−0.46	−0.17	0.12
GY	−0.70	−0.29	0.53	−0.81	0.03	−0.31	−0.20	−0.75	−0.02	−0.37	0.35
Eigenvalues	4.54	2.51	1.21	3.47	2.21	1.43	1.25	4.16	2.40	1.47	1.14
Variance (%)	37.83	20.93	10.10	28.90	18.38	11.94	10.39	34.64	20.02	12.26	9.52
Accumulated (%)	37.83	58.77	68.86	28.90	47.28	59.22	69.61	34.64	54.66	66.92	76.43

D50A, days to 50% anthesis; D50S, days to 50% silking; ASI, anthesis–silking interval; PH, plant height; EH, ear height; SPAD, chlorophyll content; EL, ear length; EG, ear girth; KRE, number of kernel rows per ear; KNR, number of kernels per a row; TW, test weight and GY, grain yield.

### 3.4 Genetic correlations

**Figure 3:**
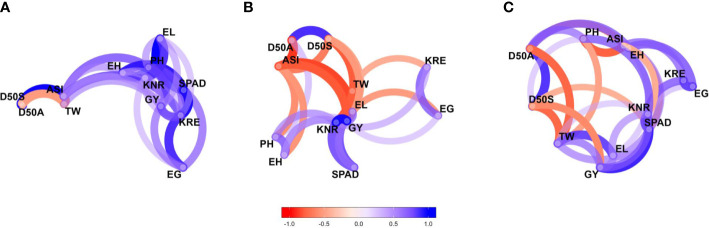
Genetic correlation network plots of 12 agronomic traits of 75 maize hybrids evaluated under **(A)** optimal, **(B)** drought, and **(C)** waterlogging during winter 2017 and summer–rainy 2018. Blue paths indicate positive correlations, and red paths indicate negative correlations. D50A, days to 50% anthesis; D50S, days to 50% silking; ASI, anthesis–silking interval; PH, plant height; EH, ear height; SPAD, chlorophyll content; EL, ear length; EG, ear girth; KRE, number of kernel rows per ear; KNR, number of kernels per a row; TW, test weight and GY, grain yield.

The pairwise correlation coefficients (*r*-values) for studied traits were estimated under each moisture regime. The network plots (**Figure 3**) of the correlation data frames of each moisture regime representing more highly associated traits appear to cluster together and connected by stronger paths where blue colored paths indicated positive correlations while red indicated negative correlations. The proximity of traits in the plots was determined by using multidimensional clustering. Inter-trait relationships are mostly positive and stronger under optimal moisture but did not follow the same trend under stress conditions. Grain yield showed a positively strong association with most of the traits under optimal moisture but not under stress. For example, GY had a strong positive correlation with PH (*r* = 0.63) under optimal conditions and had a weak correlation under drought (*r* = 0.27) and waterlogging (*r* = 0.17) conditions (**Supplementary Table 4**). Flowering traits such as D50A, D50S, and ASI showed negative correlation with GY across the moisture conditions.

### 3.5 Multi-trait genotype–ideotype distance index and selection gains

**Figure 4:**
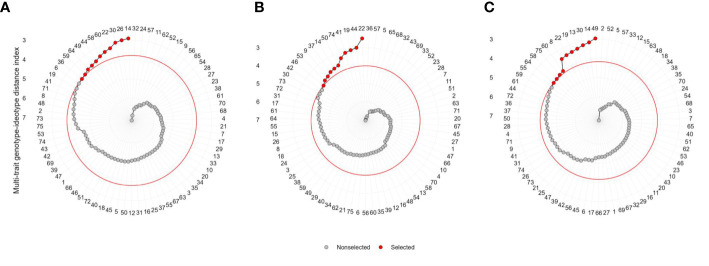
Genotype ranking in ascending order for the MGIDI index tested under **(A)** optimal, **(B)** drought, and **(C)** waterlogging. The selected hybrids are shown in red color and the red colored circle represents the cut-point according to the selection pressure (~15%).

**Figure 5:**
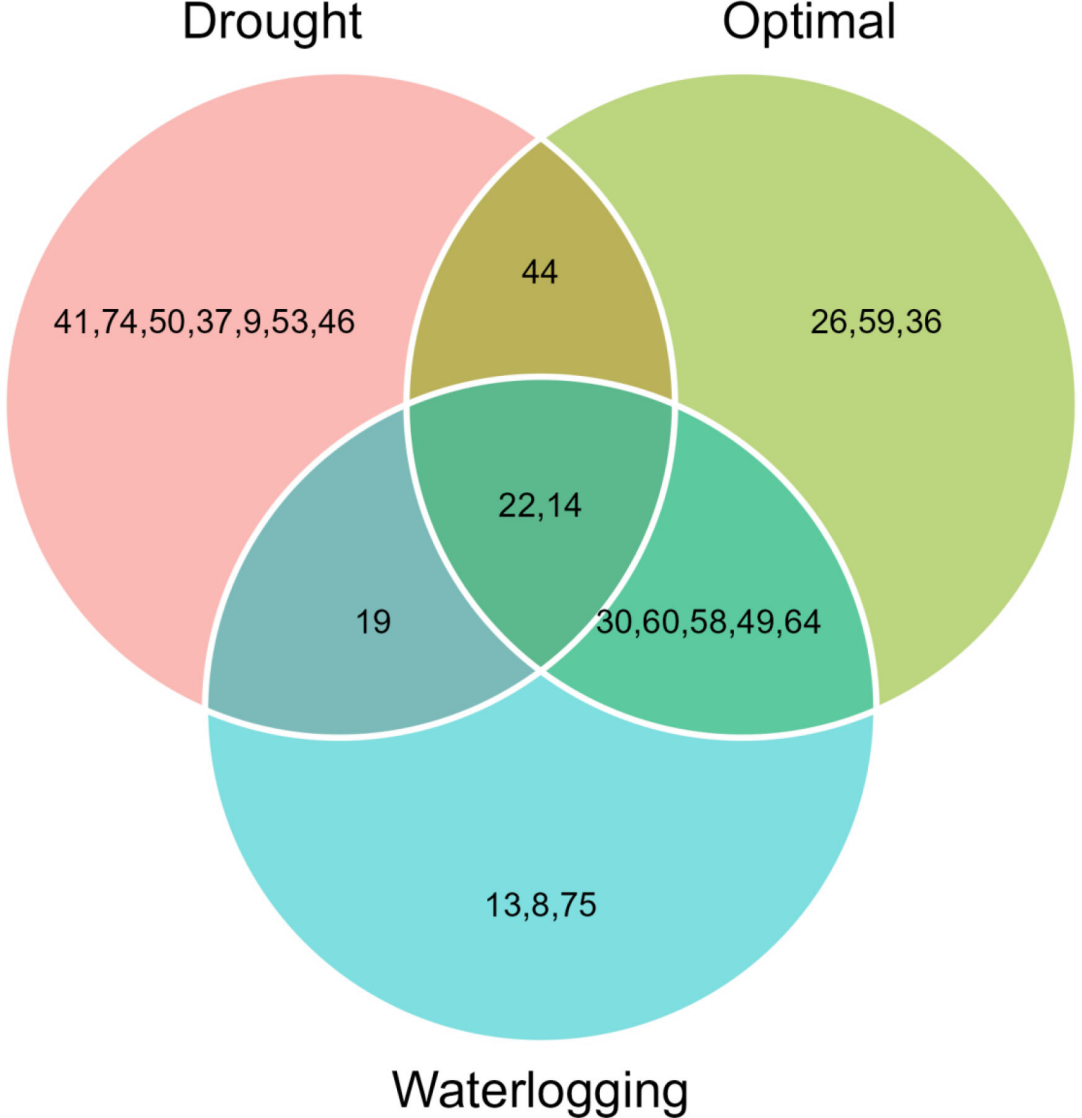
Common maize hybrid shared across the soil moisture regimes including optimal, drought, and waterlogging shown in the Venn chart.

The 11 hybrids were selected in each soil moisture condition according to the MGIDI by assuming the selection index 15% (**Table 4**). Genotypes 14 (ZH161303, MGIDI = 2.91), 26 (ZH161042, 2.97), 30 (ZH161047, 3.04), 22 (ZH161289, 3.25), 60 (ZH161129, 3.34), 58 (ZH161064, 3.45), 44 (ZH161063, 3.57), 49 (ZH161398, 3.64), 64 (ZH161078, 3.67), 59 (ZH161068, 3.76), and 36 (ZH161053, 3.79) were screened under optimal environment (**Figure 4A**), while genotypes 22 (ZH161289, 2.49), 44 (ZH161063, 2.98), 19 (ZH161458, 3.06), 41 (ZH161051, 3.13), 74 (P3502, 3.33), 50 (ZH161410, 3.63), 14 (ZH161303, 3.68), 37 (ZH161083, 3.72), 9 (ZH161384, 3.82), 53 (ZH161484, 3.87), and 46 (ZH161071, 4.01) were screened under drought (**Figure 4B**), and genotypes 49 (ZH161398), 14 (ZH161303), 30 (ZH161047), 13 (ZH161358), 19 (ZH161458), 22 (ZH161289), 8 (ZH161330), 60 (ZH161129), 75 (Hytech 5106), 58 (ZH161064), and 64 (ZH161078) were screened under waterlogging stress (**Figure 4C**). The selected hybrids under each individual moisture condition and the two hybrids, viz., 22 (ZH161289) and 14 (ZH161303), that shared in common the three moisture regimes are given in a Venn diagram (**Figure 5**, **Supplementary Table 5**).

**Table 4:** Multi-trait genotype–ideotype distance index (MGIDI) values of the 75 maize hybrids tested across seven environments during winter 2017–2018 and summer–rainy 2018.

Hybrids	MGIDI value			Hybrids	MGIDI value			Hybrid	MGIDI value		
	OPT	MDT	MWL		OPT	MDT	MWL		OPT	MDT	MWL
1	4.83	5.58	5.33	26	**2.97**	4.54	4.66	51	4.95	5.95	5.61
2	4.12	5.92	7.23	27	5.78	5.59	5.33	52	6.26	6.28	6.69
3	5.21	4.66	5.71	28	5.88	6.08	4.50	53	4.33	**3.87**	5.56
4	5.62	5.51	4.53	29	5.45	4.94	5.46	54	5.91	5.27	5.76
5	5.00	7.01	6.64	30	**3.04**	4.10	**3.22**	55	5.20	4.51	4.23
6	3.81	5.10	5.13	31	5.10	4.39	4.65	56	5.94	5.15	5.09
7	5.54	6.08	5.66	32	7.15	6.50	5.46	57	6.54	7.03	6.59
8	4.06	4.59	**3.48**	33	5.42	6.38	6.52	58	**3.45**	5.45	**4.14**
9	6.09	**3.82**	4.58	34	5.23	5.01	5.96	59	**3.76**	4.92	4.26
10	5.31	5.53	5.53	35	5.22	5.20	5.92	60	**3.34**	5.16	**4.05**
11	6.43	5.96	5.50	36	**3.79**	7.04	4.45	61	5.73	4.40	4.32
12	5.07	5.21	6.42	37	5.17	**3.72**	4.46	62	6.38	5.01	5.59
13	5.44	5.28	**3.33**	38	5.73	4.91	5.92	63	5.21	5.78	6.19
14	**2.91†**	**3.68**	**3.13**	39	4.68	5.21	4.90	64	**3.67**	4.44	**4.16**
15	6.24	4.53	6.24	40	4.98	4.95	5.62	65	5.92	6.89	5.63
16	5.11	5.22	5.49	41	3.88	**3.13**	4.59	66	4.93	5.54	5.32
17	5.46	4.40	5.26	42	4.65	4.09	4.94	67	5.20	5.65	5.41
18	4.98	4.60	5.97	43	4.64	6.45	5.52	68	5.63	6.79	5.74
19	3.85	**3.06**	**3.39**	44	**3.57**	**2.98**	4.37	69	4.67	6.44	5.40
20	5.27	5.66	5.51	45	4.99	5.65	5.11	70	5.67	5.46	5.89
21	5.58	5.07	4.86	46	4.94	**4.01**	5.56	71	3.98	5.72	4.56
22	**3.25**	**2.49**	**3.43**	47	4.79	5.56	4.87	72	4.96	4.31	4.41
23	5.77	6.14	5.54	48	4.07	5.23	5.98	73	4.16	4.12	4.75
24	6.89	4.65	5.79	49	**3.64**	4.92	**2.95**	74	4.60	**3.33**	4.66
25	5.16	4.82	4.87	50	5.02	**3.63**	4.46	75	4.24	5.07	**4.10**

MGIDI, multi-trait genotype–ideotype distance index; OPT, optimal; MDT, managed drought; MWL, managed waterlogging.

†Bold values indicate selected hybrids by assuming 15% selection intensity.

The selected genotypes under each moisture regime resulted in desired selection gains (SGs) for the mean performance of all the studied traits, i.e., positive SGs for GY, PH, EH, EG, EL, KNR, KRE, and SPAD, and negative gains for the flowering traits such as D50A, D50S, and ASI (**Table 5**). The mean performance of selected genotypes for GY increased more than 11% SG under optimal and drought conditions, whereas it was 22.6% under waterlogging. The contribution of each factor retained towards the distance from MGIDI to the ideotype (ID) under the three moisture regimes is shown in **Supplementary Table 6**.

**Table 5:** Selection gains for mean performance of 75 maize hybrids within the soil moisture regime based on MGIDI values.

Trait†	Factor	Sense	Goal	Mean performance			
				Xo	Xs	SD	SD%
Optimal							
PH	FA1	Increase	Yes	170.96	174.14	3.18	1.86
EH	FA1	Increase	Yes	90.66	92.67	2.01	2.22
SPAD	FA1	Increase	Yes	48.30	49.05	0.75	1.55
EL	FA1	Increase	Yes	18.26	18.63	0.37	2.02
KNR	FA1	Increase	Yes	33.73	34.22	0.49	1.46
GY	FA1	Increase	Yes	7.87	8.74	0.87	11.09
D50A	FA2	Decrease	Yes	67.18	66.18	−1.00	−1.48
D50S	FA2	Decrease	Yes	69.60	68.59	−1.02	−1.46
ASI	FA2	Decrease	Yes	2.42	2.42	−0.01	−0.23
TW	FA2	Increase	Yes	29.05	29.82	0.77	2.65
EG	FA3	Increase	Yes	4.34	4.41	0.07	1.58
KRE	FA3	Increase	Yes	14.80	15.20	0.40	2.70
Managed Drought							
TW	FA1	Increase	Yes	25.36	25.28	0.08	0.32
EL	FA1	Increase	Yes	16.79	17.13	0.34	2.05
KNR	FA1	Increase	Yes	31.82	33.13	1.31	4.11
GY	FA1	Increase	Yes	4.39	4.90	0.51	11.67
D50A	FA2	Decrease	Yes	89.30	88.47	−0.83	−0.93
D50S	FA2	Decrease	Yes	95.00	94.48	−0.52	−0.54
ASI	FA2	Decrease	Yes	5.71	5.58	−0.13	−2.28
EG	FA3	Increase	Yes	3.99	4.04	0.05	1.17
KRE	FA3	Increase	Yes	14.72	14.98	0.26	1.76
PH	FA4	Increase	Yes	151.36	153.83	2.47	1.63
EH	FA4	Increase	Yes	91.25	96.72	5.47	6.00
SPAD	FA4	Increase	Yes	39.14	40.22	1.09	2.77
Managed Waterlogging							
SPAD	FA1	Increase	Yes	25.04	28.52	3.48	13.90
TW	FA1	Increase	Yes	25.10	25.62	0.52	2.07
EL	FA1	Increase	Yes	13.92	14.25	0.33	2.40
KNR	FA1	Increase	Yes	25.84	27.31	1.47	5.68
GY	FA1	Increase	Yes	3.80	4.65	0.86	22.59
D50A	FA2	Decrease	Yes	56.33	55.97	−0.36	−0.64
PH	FA2	Increase	Yes	138.49	141.44	2.95	2.13
EH	FA2	Increase	Yes	76.56	78.60	2.03	2.66
EG	FA3	Increase	Yes	3.93	4.04	0.11	2.79
KRE	FA3	Increase	Yes	14.23	14.60	0.37	2.61
D50S	FA4	Decrease	Yes	61.23	60.74	−0.48	−0.79
ASI	FA4	Decrease	Yes	4.89	4.69	−0.20	−4.06

FA1, factor 1; FA2, factor 2; FA3, factor 3; FA4, factor 4; †D50A, days to 50% anthesis; D50S, days to 50% silking; ASI, anthesis–silking interval; PH, plant height; EH, ear height; SPAD, chlorophyll content; EL, ear length; EG, ear girth; KRE, number of kernel rows per ear; KNR, number of kernels per a row; TW, test weight and GY, grain yield; SD, selection differential; SD%, percent selection differential; Xo, mean of the original population; Xs, mean of the selected hybrids.

### 3.6 The strengths and weaknesses view of selected hybrids

**Figure 6:**
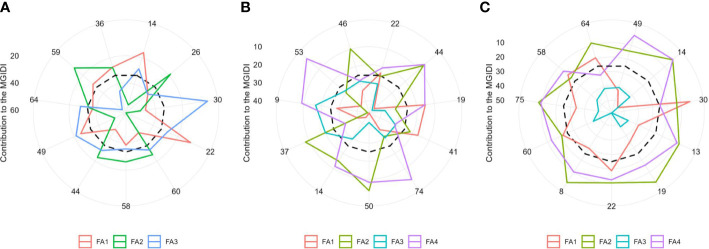
The strengths and weaknesses view of the selected hybrids is shown as the proportion of each factor on the computed MGIDI values over three soil moisture regimes, namely, **(A)** optimal, **(B)** drought, and **(C)** waterlogging. The smallest the proportion explained by a factor (closer to the external edge), the closer the traits within that factor are to the ideotype. The dashed line shows the theoretical value if all the factors had contributed equally.

The radar plot (**Figures 6A–C**) depicts the strengths and weaknesses of the selected hybrids over three moisture conditions. For each selected hybrid, the contribution of each factor towards the MGIDI is ranked from the most contributing factor (close to plot center) to the least contributing factor (away from the plot center). Smaller proportions explained by a factor that is placed closer to the external edge indicate that the trait within that factor is more similar to the ideotype. A view on strengths and weaknesses under optimal moisture revealed that the performance of the selected genotypes, viz., 22 (ZH161289) and 14 (ZH161303), showed strengths related to factor FA1 that holds PH, EH, SPAD, EL, KNR, and GY with positive SGs, whereas genotypes 59 (ZH161068), 26 (ZH161042), 44 (ZH161063), 58 (ZH161064), and 60 (ZH161129) showed strengths related to FA2 with flowering characteristics (D50A, D50S, and ASI) showing negative gains (**Figure 6A**). Concerning FA3, genotypes 30 (ZH161047) and 49 (ZH161398) performed well. Under managed drought (**Figure 6B**), most of the selected hybrids contributed more towards MGIDI through FA1 except 19 (ZH161458) and 41 (ZH161051). Hybrids 50 (ZH161410), 44 (ZH161063), 37 (ZH161083), and 46 (ZH161071) showed strength related to FA2 with desirable negative gain in flowering traits such as D50A, D50S, and ASI. Genotypes 9 (ZH161384) and 46 (ZH161071) had strength for FA3 whereas 53 (ZH161484) followed by 74 (P3502), 44 (ZH161063), 50 (ZH161410), and 9 (ZH161384) showed strengths pertaining to FA4. Similarly under waterlogging conditions (**Figure 6C**), most of the selected genotypes showed strengths related to all the factors except FA3. According to FA1, genotype 30 (ZH161047) performed very well while genotype 14 (ZH161303) performed poorly with a maximum contribution towards the MGIDI. All the 11 selected hybrids showed strengths related to FA2 and FA3 and weaknesses related to FA3.

## 4 Discussion

The study focused on the performance evaluation of 75 pre-released medium-duration maize hybrids for 12 agronomic characteristics in two consecutive years (2017–2018) under three soil moisture regimes including optimal, drought, and waterlogging at two locations. In general, the grain yield potential and the performance of other yield-contributing characteristics of the available germplasm are a very crucial step in identifying genotypes with ideal trait combinations suitable across target environments, which can be used in future breeding programs (Bocianowski et al., 2019). The maize crop in the farmer’s field is exposed to a combination of stresses in a single life cycle. Banziger et al. (2000) screened a set of tropical maize hybrids independently under drought and low N environments and identified several physiological traits associated with tolerance under a single stress and conferred tolerance for the other stress. Our results showed that the environment and GEI effects for most of the traits recorded were statistically significant (*p* ≤ 0.001 or *p* ≤ 0.01). This shows that hybrid mean performances varied across the soil moisture conditions, which can be attributed more towards the diversification of genotypes and provides ample amount of variation for easy selection (Oliveira et al., 2014). The results from the present investigation suggested that selection of maize hybrids based on multi-factorial analysis is effective and that most of the potential and wide adoptive maize hybrids were influenced by soil moisture status and genetic factors, as well as their interactions (GEI). Understanding the complexity of GEI through suitable statistical techniques is the best way to screen out the wide adoptable stress-resilient maize hybrids in the target environment. Statistically significant variation of G, E, and GEI among evaluated hybrids across the moisture regimes would make it possible to identify genotypes for both stress and non-stress environments. Previous works also reported the existence of significant variations among maize hybrids tested across managed stresses for grain yield (Wegary et al., 2014; Abakemal et al., 2016; Njeri et al., 2017; Adeseye et al., 2018; Mebratu et al., 2019; Singamsetti et al., 2021).

Since the physiological and molecular responses of crop plants exposed to a combination of stresses are fairly known, understanding the effects of different individual stresses on crop plants as well as their combinations is vital (Voesenek and Pierik, 2008). Early flowering (D50A and D50S), high ASI values, and lower mean performances for PH, EH, TW, EL, EG, NKE, and NKR under moisture stress conditions compared to optimal environments resulted in poor grain yields (**Supplementary Tables 2A–C**). The impact of moisture level on traits’ expression can be observed by their mean performances under different environments (**Figure 1**). A few outliers in the box plots of recorded traits were probably due to inconsistent expressions that indicated the severity of stress and interaction of genotype with soil moisture level (**Figure 1**). Delayed flowering and maturity due to cool temperature at early stages followed by prolonged day length and drought periods at later stages resulted in the drastic decline (up to 30%–90%) in the crop yield in late *Rabi*/winter maize crop (Sah et al., 2020). Our study also witnessed early senescence probably due to the reduction in lamellar content of light harvest chlorophyll proteins under water-limited situations (Anjum et al., 2011; Verma et al., 2004). The observed significant reduction in mean GY under waterlogging conditions with a poor performance of other secondary traits is probably due to the reduced leaf growth, and a worse effect on the cell turgor could be attributed to the decline in the carotenoid content (El-Shihaby et al., 2002; Tripathi et al., 2003).

Understanding the magnitude and direction of correlations among studied traits and how they change across the moisture regimes along with variability parameters would assist the breeders in improving a characteristic that brings simultaneous improvement in other characteristics. The results explained the importance of flowering traits, especially ASI, in the development of drought-tolerant maize cultivars. The larger ASI values lead to the failure of a proper seed set due to poor availability of viable pollen for late emerged female flowers owing to the delay in silk extrusion, premature lodging, and reduced rates of net photosynthesis arising from oxidative damage to chloroplasts (Nelimor et al., 2020). The relationship between GY and ASI in our findings was in agreement with previous reports on maize under water deficit (Edmeades and Daynard, 1979) and under waterlogging conditions (Zaidi and Singh, 2001; Zaidi et al., 2004; Zaidi et al., 2007). The stronger associations between grain yield and phenological traits under drought conditions than under optimal moisture were reported by Sah et al. (2020). In contrast, our results suggested that the direct selection of most of the traits would be more effective under optimal conditions due to the resulting strong correlation, while there is only moderate correlation under moisture stress conditions (**Figure 3**). Several previous correlation studies on maize genotypes under a single environment have been reported, but there is limited literature available with regard to changing a trait’s expression and the association of a trait with other traits across various environments. A few researchers reported genetic correlations among maize lines evaluated across two moisture regimes, namely, moisture-deficit and well-water conditions, across two seasons/years (Oyekunle and Badu-Apraku, 2018; Nelimor et al., 2019; Al-Naggar et al., 2020; Nelimor et al., 2020; Singamsetti et al., 2021).

Plant breeding programs aiming at initial selection under a single environment with no stress or only a single stress condition may lead to the loss of desirable alleles or genetic variability for additional stresses other than target stress (Cairns et al., 2013). Thus, the use of the MGIDI becomes especially important for screening hybrids based on multiple traits such as many secondary traits along with grain yield under various stress environments. Apart from many classical linear multi-trait phenotypic selection techniques available (Smith, 1936; Hazel, 1943; Bhering et al., 2012; Jahufer and Casler, 2015; Bizari et al., 2017; Cerón-Rojas and Crossa, 2018; Burdon and Li, 2019), MGIDI is a unique selection technique that is free from weighing (economic) coefficients (Bizari et al., 2017) and multicollinearity issues (Smith, 1936), and it uses the distance between genotypes and a defined ideotype based on the breeder’s requirement (Olivoto and Nardino, 2021). The method emerged as a powerful tool to identify the genotypes with better mean performance and desired SGs and also to estimate the strengths and weaknesses of hybrids (selected/unselected). The multi-trait selection index (MTSI) and MGIDI evaluation systems are novel and unique techniques that have many practical applications in plant breeding practices (Nelimor et al., 2020; Benakanahalli et al., 2021; Koundinya et al., 2021; Pour-Aboughadareh et al., 2021; Hadou el hadj et al., 2022; Yue et al., 2022a; Yue et al.,2022b; Nardino et al., 2022; Dhand and Garg, 2023; Memon et al., 2023). The multi-trait frameworks and MGIDI and MTSI indices follow a similar rescaling process to retain the unchanged correlation structure of original data and to identify the superior genotypes with respect to multiple traits simultaneously (Olivoto et al., 2019b). The rescaling technique requires a selection direction, and it places all the agronomic traits in a range of 0–100 and the ideotype defined by traits’ rescaled value is assumed to be 100. The dimensional reduction was carried out by performing exponential factorial analysis in which 12 traits represented a few (three in optimal and four each in drought and waterlogging) final latent variables (FAs, factors) with maximum trait loadings.

According to the MGIDI index, the selected genotypes in optimal moisture were ZH161303, ZH161042, ZH161047, ZH161289, ZH161129, ZH161064, ZH161063, ZH161398, ZH161078, ZH161068, and ZH161053; the selected genotypes under drought conditions were ZH161289, ZH161063, ZH161458, ZH161051, P3502, ZH161410, ZH161303, ZH161083, ZH161384, ZH161484, and ZH161071; and the selected genotypes under waterlogging stress were ZH161398, ZH161303, ZH161047, ZH161358, ZH161458, ZH161289, ZH161330, ZH161129, Hytech 5106, ZH161064, and ZH161078. Apart from the genotypes selected above, genotypes 6 (ZH161361), 42 (ZH161054), and 55 (ZH15449) were placed at the cutting points under optimal, drought, and waterlogging conditions, respectively (**Figure 4**). These hybrids had interesting features that could be exploited in future breeding programs. The MGIDI index is a unique and easy-to-interpret selection procedure that has many practical applications to obtain long-term genetic gain in primary traits (such as grain yield) without jeopardizing gains of secondary traits (such as plant height). The proportion explained by each factor towards the MGIDI index, i.e., “strengths and weaknesses view” (**Figures 6A–C**), is an important graphical tool to identify the strengths and weaknesses of test hybrids in terms of “trait (group of traits) need to be improved” and is an added advantage over existing indices. For example, under optimal moisture, the lower contribution of FA1 in genotype 14 (ZH161303) revealed that the genotype was highly productive in terms of PH, EH, SPAD, EL, KNR, and GY, but the same genotype had a poor performance in terms of flowering traits, which can be inferred due to the higher contribution of FA2 towards MGIDI (**Figure 6A**). Similarly, FA3 was placed almost near the dashed lines of the radar plot, which represented the theoretical value if all the factors had contributed equally. The high contribution of FA1 (**Figure 6B**) and FA3 (**Figure 6C**) towards genotype–ideotype index distance indicated that there is a possibility of improvement in TW, EL, KNR, and GY in selected genotypes (except 19 and 41) under drought and in EG and KRE under waterlogging conditions. A similar study was reported by Gabriel et al. (2019) and Olivoto and Nardino (2021), who evaluated a set of 13 strawberry cultivars, wherein the strengths were described by employing MGIDI. Benakanahalli et al. (2021) proposed a framework for identifying promising guar genotypes with productive traits such as gum and seed yield across three seasons by using MGIDI.

## 5 Conclusion

Our experimental findings recommended that MGIDI can be used for the effective selection of superior hybrids/genotypes by considering multiple traits and helping plant breeders make better strategic decisions. The results showed that the hybrids ZH161053, ZH161289, ZH161063, ZH161398, and ZH161047 were found to be common across all the three moisture regimes solely on the basis of grain yield, while two genotypes, ZH161289 and ZH161303, were found to be common in terms of all the studied traits. Also, the study helps improve a certain trait or a group of traits (either yield contributors or stress-responsive ones) in particular moisture stress environments.

## Data availability statement

The original contributions presented in the study are included in the article/**Supplementary Material**. Further inquiries can be directed to the corresponding authors.

## Author contributions

AS, PZ, and KSe contributed to the conceptualization and design of the study, field experimentation, and preparation of the manuscript. KSe, MV, and AM revised and edited the original manuscript. AS and TV contributed to formal analysis of data and visualization. AS, KM, MK and KSh contributed to field experiments and data recording. All authors have read and approved the final manuscript.
