# Supplementary material for: Genetic gains in tropical maize hybrids across moisture regimes with multi-trait-based index selection
Source: Front Plant Sci. 2023 Mar 3;14:1147424. doi: 10.3389/fpls.2023.1147424 (PMC10020505; doi:10.3389/fpls.2023.1147424)
Supplement: Supplementary file 1 [file DataSheet_1.docx]

**Supplemental Table 1. List of 75 maize hybrids evaluated in seven test environments including three moisture regimes during winter 2017 and summer-rainy 2018**

| **Genotype code** | **Name of the genotype** | **Genotype code** | **Name of the genotype** | **Genotype code** | **Name of the genotype** |
| --- | --- | --- | --- | --- | --- |
| **1** | ZH161271 | **26** | ZH161042 | **51** | ZH161478 |
| **2** | ZH161475 | **27** | ZH161079 | **52** | ZH161456 |
| **3** | ZH161493 | **28** | ZH161076 | **53** | ZH161484 |
| **4** | ZH161464 | **29** | ZH161039 | **54** | ZH161463 |
| **5** | ZH161409 | **30** | ZH161047 | **55** | ZH15449 |
| **6** | ZH161361 | **31** | ZH161100 | **56** | ZH161196 |
| **7** | ZH161485 | **32** | ZH161045 | **57** | ZH161194 |
| **8** | ZH161330 | **33** | ZH161095 | **58** | ZH161064 |
| **9** | ZH161384 | **34** | ZH161060 | **59** | ZH161068 |
| **10** | ZH161418 | **35** | ZH161054 | **60** | ZH161129 |
| **11** | ZH161276 | **36** | ZH161053 | **61** | ZH161077 |
| **12** | ZH161311 | **37** | ZH161083 | **62** | ZH161066 |
| **13** | ZH161358 | **38** | ZH161120 | **63** | ZH161137 |
| **14** | ZH161303 | **39** | ZH161038 | **64** | ZH161078 |
| **15** | ZH161438 | **40** | ZH161054 | **65** | ZH161114 |
| **16** | ZH161382 | **41** | ZH161051 | **66** | ZH161043 |
| **17** | ZH161434 | **42** | ZH161054 | **67** | ZH161184 |
| **18** | ZH161377 | **43** | ZH161082 | **68** | ZH161050 |
| **19** | ZH161458 | **44** | ZH161063 | **69** | ZH161207 |
| **20** | ZH161452 | **45** | ZH161093 | **70** | ZH161089 |
| **21** | ZH161473 | **46** | ZH161071 | **71** | CAH 153 |
| **22** | ZH161289 | **47** | ZH161210 | **72** | CAH 1511 |
| **23** | ZH161471 | **48** | ZH161102 | **73** | MG 900 |
| **24** | ZH161285 | **49** | ZH161398 | **74** | P3502 |
| **25** | ZH161135 | **50** | ZH161410 | **75** | Hytech 5106 |

**Supplemental table 2a. Mean performance for recorded traits of 75 maize hybrids under optimal moisture condition**

| **Code** | **Genotype** | **D50A** | **D50S** | **ASI** | **PH** | **EH** | **SPAD** | **TW** | **EL** | **EG** | **KRE** | **KNR** | **GY** |
| --- | --- | --- | --- | --- | --- | --- | --- | --- | --- | --- | --- | --- | --- |
| 1 | ZH161271 | 66.83 | 69.83 | 3.00 | 183.83 | 92.33 | 51.78 | 29.60 | 17.43 | 4.33 | 14.24 | 31.13 | 8.25 |
| 2 | ZH161475 | 66.50 | 68.50 | 2.00 | 167.67 | 95.83 | 49.11 | 29.41 | 17.63 | 4.47 | 14.93 | 34.36 | 8.92 |
| 3 | ZH161493 | 69.00 | 71.33 | 2.33 | 174.17 | 97.00 | 50.46 | 26.94 | 18.23 | 4.34 | 14.85 | 35.05 | 8.15 |
| 4 | ZH161464 | 69.67 | 71.50 | 1.83 | 171.33 | 92.17 | 44.83 | 27.53 | 17.73 | 4.36 | 14.35 | 33.69 | 7.90 |
| 5 | ZH161409 | 66.67 | 69.67 | 3.00 | 172.00 | 99.00 | 49.91 | 28.18 | 18.27 | 4.10 | 14.29 | 35.08 | 7.91 |
| 6 | ZH161361 | 67.67 | 70.50 | 2.83 | 194.17 | 112.50 | 53.07 | 28.72 | 19.50 | 4.93 | 18.33 | 36.85 | 9.87 |
| 7 | ZH161485 | 69.67 | 71.83 | 2.17 | 162.67 | 90.83 | 46.25 | 26.45 | 18.47 | 4.54 | 15.10 | 34.10 | 7.65 |
| 8 | ZH161330 | 68.33 | 70.50 | 2.17 | 176.17 | 97.50 | 54.57 | 28.29 | 18.98 | 4.75 | 14.66 | 36.63 | 8.94 |
| 9 | ZH161384 | 70.17 | 72.50 | 2.33 | 180.83 | 103.33 | 47.01 | 27.32 | 19.51 | 4.34 | 14.37 | 35.33 | 6.83 |
| 10 | ZH161418 | 70.83 | 73.00 | 2.17 | 188.50 | 101.67 | 46.62 | 29.88 | 17.91 | 4.40 | 15.09 | 35.58 | 8.79 |
| 11 | ZH161276 | 67.83 | 70.33 | 2.50 | 172.17 | 95.83 | 43.42 | 24.99 | 16.76 | 4.00 | 14.17 | 34.61 | 6.93 |
| 12 | ZH161311 | 67.50 | 70.00 | 2.50 | 175.67 | 99.82 | 50.81 | 28.07 | 18.34 | 4.36 | 13.72 | 35.84 | 7.33 |
| 13 | ZH161358 | 68.00 | 70.17 | 2.17 | 172.33 | 94.67 | 48.73 | 26.77 | 17.04 | 4.43 | 14.65 | 31.28 | 7.70 |
| 14 | ZH161303 | 66.17 | 68.50 | 2.33 | 196.67 | 117.50 | 54.22 | 30.60 | 18.87 | 4.77 | 16.09 | 37.26 | 9.85 |
| 15 | ZH161438 | 70.83 | 73.83 | 3.00 | 173.17 | 97.17 | 51.54 | 28.07 | 17.81 | 4.36 | 14.25 | 33.70 | 6.53 |
| 16 | ZH161382 | 67.50 | 69.67 | 2.17 | 162.00 | 90.17 | 48.54 | 26.58 | 17.72 | 4.57 | 15.15 | 32.60 | 7.69 |
| 17 | ZH161434 | 66.67 | 69.67 | 3.00 | 183.00 | 102.83 | 45.50 | 27.29 | 17.43 | 4.30 | 15.13 | 32.89 | 6.81 |
| 18 | ZH161377 | 64.33 | 66.50 | 2.17 | 164.33 | 93.33 | 47.50 | 28.09 | 18.51 | 4.02 | 14.12 | 33.94 | 7.75 |
| 19 | ZH161458 | 66.83 | 69.67 | 2.83 | 177.50 | 92.67 | 51.46 | 28.29 | 19.45 | 4.40 | 14.90 | 38.49 | 9.77 |
| 20 | ZH161452 | 68.50 | 70.33 | 1.83 | 175.00 | 102.50 | 44.66 | 26.84 | 17.21 | 4.35 | 15.09 | 35.09 | 7.84 |
| 21 | ZH161473 | 69.67 | 72.50 | 2.83 | 165.00 | 94.17 | 44.46 | 29.19 | 17.77 | 4.36 | 14.89 | 35.82 | 7.79 |
| 22 | ZH161289 | 67.33 | 70.17 | 2.83 | 190.83 | 111.67 | 61.08 | 29.88 | 19.01 | 4.42 | 16.11 | 38.60 | 10.48 |
| 23 | ZH161471 | 66.67 | 69.00 | 2.33 | 161.00 | 86.67 | 49.03 | 27.50 | 16.16 | 4.25 | 13.94 | 31.27 | 6.89 |
| 24 | ZH161285 | 69.67 | 72.50 | 2.83 | 153.33 | 89.17 | 43.01 | 26.84 | 17.49 | 4.32 | 13.84 | 31.13 | 5.47 |
| 25 | ZH161135 | 67.00 | 68.50 | 1.50 | 175.17 | 83.33 | 48.32 | 29.83 | 17.02 | 4.22 | 14.14 | 31.52 | 7.41 |
| 26 | ZH161042 | 65.83 | 67.33 | 1.50 | 171.00 | 80.00 | 51.67 | 30.97 | 19.81 | 4.61 | 15.90 | 36.47 | 9.10 |
| 27 | ZH161079 | 68.17 | 72.33 | 4.17 | 168.33 | 77.50 | 44.84 | 29.67 | 17.95 | 4.26 | 14.76 | 32.76 | 8.08 |
| 28 | ZH161076 | 66.00 | 68.67 | 2.67 | 150.17 | 73.33 | 46.86 | 26.39 | 17.19 | 4.23 | 14.65 | 31.31 | 6.84 |
| 29 | ZH161039 | 64.67 | 67.00 | 2.33 | 167.50 | 84.17 | 44.05 | 26.63 | 17.18 | 4.21 | 14.49 | 31.63 | 6.98 |
| 30 | ZH161047 | 66.17 | 69.50 | 3.33 | 177.50 | 95.00 | 53.97 | 32.62 | 19.38 | 4.72 | 16.17 | 37.47 | 10.35 |
| 31 | ZH161100 | 66.33 | 68.50 | 2.17 | 165.83 | 84.17 | 49.26 | 28.90 | 17.71 | 4.22 | 14.03 | 32.26 | 7.50 |
| 32 | ZH161045 | 69.33 | 72.33 | 3.00 | 154.17 | 75.00 | 42.19 | 27.78 | 17.38 | 3.88 | 14.05 | 29.59 | 5.82 |
| 33 | ZH161095 | 66.50 | 69.33 | 2.83 | 152.67 | 75.83 | 45.71 | 28.36 | 17.34 | 4.49 | 14.67 | 34.20 | 7.45 |
| 34 | ZH161060 | 66.00 | 68.00 | 2.00 | 168.33 | 82.50 | 42.98 | 29.36 | 18.59 | 4.21 | 14.30 | 31.02 | 7.41 |
| 35 | ZH161054 | 67.17 | 69.00 | 1.83 | 184.83 | 96.83 | 46.62 | 30.29 | 18.57 | 4.01 | 14.22 | 34.30 | 7.83 |
| 36 | ZH161053 | 66.17 | 68.17 | 2.00 | 184.17 | 88.00 | 49.79 | 31.60 | 19.38 | 4.24 | 14.97 | 35.07 | 9.24 |
| 37 | ZH161083 | 64.50 | 66.50 | 2.00 | 163.83 | 83.33 | 44.08 | 28.40 | 17.39 | 4.36 | 15.03 | 30.78 | 6.82 |
| 38 | ZH161120 | 67.33 | 70.33 | 3.00 | 155.17 | 75.83 | 46.44 | 28.54 | 17.73 | 4.12 | 15.62 | 31.55 | 7.08 |
| 39 | ZH161038 | 66.83 | 69.50 | 2.67 | 163.83 | 93.00 | 48.89 | 29.21 | 19.30 | 4.19 | 14.58 | 35.25 | 7.92 |
| 40 | ZH161054 | 65.67 | 68.50 | 2.83 | 157.50 | 74.17 | 48.21 | 31.14 | 18.28 | 4.33 | 14.25 | 31.80 | 7.56 |
| 41 | ZH161051 | 67.50 | 70.00 | 2.50 | 177.33 | 91.67 | 49.11 | 30.76 | 20.05 | 4.37 | 15.51 | 35.55 | 8.71 |
| 42 | ZH161054 | 66.67 | 68.50 | 1.83 | 169.17 | 87.50 | 48.78 | 30.51 | 18.81 | 4.38 | 14.12 | 33.75 | 7.65 |
| 43 | ZH161082 | 65.00 | 66.67 | 1.67 | 160.50 | 82.67 | 43.58 | 30.68 | 17.80 | 4.52 | 14.52 | 32.45 | 8.15 |
| 44 | ZH161063 | 64.33 | 66.83 | 2.50 | 174.50 | 90.83 | 50.15 | 31.15 | 17.70 | 4.43 | 15.02 | 35.07 | 9.17 |
| 45 | ZH161093 | 67.00 | 69.50 | 2.50 | 166.67 | 88.00 | 44.32 | 29.02 | 18.62 | 4.32 | 14.53 | 35.06 | 7.39 |
| 46 | ZH161071 | 65.50 | 67.83 | 2.33 | 163.50 | 80.83 | 50.27 | 30.87 | 18.43 | 4.13 | 13.94 | 31.21 | 7.39 |
| 47 | ZH161210 | 67.17 | 70.00 | 2.83 | 179.83 | 85.83 | 50.25 | 30.09 | 17.61 | 4.76 | 17.45 | 32.47 | 8.60 |
| 48 | ZH161102 | 62.67 | 64.67 | 2.00 | 170.00 | 92.67 | 46.73 | 31.06 | 18.01 | 4.27 | 14.95 | 33.60 | 8.36 |
| 49 | ZH161398 | 66.33 | 69.67 | 3.33 | 191.33 | 103.33 | 52.09 | 29.44 | 19.12 | 4.46 | 15.91 | 36.69 | 9.63 |
| 50 | ZH161410 | 66.17 | 68.67 | 2.50 | 168.67 | 99.17 | 47.37 | 25.82 | 18.65 | 4.50 | 14.80 | 33.79 | 7.22 |
| 51 | ZH161478 | 68.33 | 70.67 | 2.33 | 165.17 | 83.50 | 47.69 | 30.91 | 17.20 | 5.06 | 15.47 | 33.34 | 8.17 |
| 52 | ZH161456 | 70.83 | 73.00 | 2.17 | 176.00 | 93.33 | 45.05 | 28.06 | 19.29 | 4.07 | 14.26 | 34.48 | 7.21 |
| 53 | ZH161484 | 68.17 | 70.67 | 2.50 | 183.50 | 106.67 | 50.18 | 30.36 | 18.73 | 4.56 | 15.76 | 35.13 | 7.89 |
| 54 | ZH161463 | 66.67 | 69.17 | 2.50 | 151.67 | 85.00 | 48.22 | 27.10 | 16.95 | 4.05 | 13.94 | 31.04 | 6.81 |
| 55 | ZH15449 | 67.83 | 69.83 | 2.00 | 185.33 | 99.17 | 45.49 | 29.27 | 18.52 | 4.18 | 14.11 | 34.95 | 8.23 |
| 56 | ZH161196 | 70.83 | 73.50 | 2.67 | 172.67 | 94.83 | 47.71 | 30.01 | 18.34 | 3.98 | 14.58 | 31.56 | 8.26 |
| 57 | ZH161194 | 70.67 | 73.17 | 2.50 | 164.17 | 84.00 | 47.83 | 29.94 | 17.07 | 4.08 | 13.71 | 29.89 | 6.61 |
| 58 | ZH161064 | 65.50 | 67.50 | 2.00 | 175.17 | 91.67 | 50.84 | 31.40 | 19.45 | 4.56 | 15.18 | 33.74 | 8.66 |
| 59 | ZH161068 | 64.50 | 66.33 | 1.83 | 171.00 | 80.83 | 51.21 | 30.08 | 20.57 | 4.28 | 14.76 | 36.44 | 8.90 |
| 60 | ZH161129 | 65.83 | 67.83 | 2.00 | 168.83 | 86.50 | 50.05 | 31.22 | 18.62 | 4.52 | 15.68 | 34.72 | 9.29 |
| 61 | ZH161077 | 67.17 | 69.33 | 2.17 | 172.50 | 94.17 | 47.52 | 29.19 | 18.66 | 3.94 | 14.20 | 30.49 | 6.67 |
| 62 | ZH161066 | 65.50 | 68.50 | 3.00 | 151.67 | 80.00 | 44.11 | 29.10 | 16.71 | 4.38 | 14.76 | 27.58 | 5.48 |
| 63 | ZH161137 | 66.83 | 68.83 | 2.00 | 170.83 | 84.17 | 48.75 | 29.80 | 19.33 | 4.08 | 13.65 | 34.78 | 7.37 |
| 64 | ZH161078 | 67.67 | 69.83 | 2.17 | 173.67 | 82.50 | 54.31 | 31.40 | 19.04 | 4.52 | 15.26 | 33.85 | 8.98 |
| 65 | ZH161114 | 68.50 | 71.83 | 3.33 | 179.17 | 94.33 | 43.92 | 26.55 | 18.26 | 4.46 | 14.76 | 33.04 | 7.26 |
| 66 | ZH161043 | 66.00 | 68.83 | 2.83 | 176.67 | 84.33 | 46.05 | 29.56 | 17.84 | 4.18 | 14.01 | 32.94 | 8.07 |
| 67 | ZH161184 | 65.33 | 67.00 | 1.67 | 156.67 | 77.00 | 47.17 | 29.07 | 17.70 | 4.29 | 14.14 | 32.26 | 6.83 |
| 68 | ZH161050 | 69.50 | 72.83 | 3.33 | 172.50 | 93.33 | 48.03 | 28.90 | 18.63 | 4.38 | 15.54 | 33.90 | 7.34 |
| 69 | ZH161207 | 66.67 | 69.00 | 2.33 | 158.33 | 84.17 | 49.22 | 28.79 | 19.21 | 4.34 | 14.69 | 32.79 | 7.34 |
| 70 | ZH161089 | 67.83 | 70.33 | 2.50 | 142.67 | 75.17 | 47.02 | 29.97 | 17.97 | 4.01 | 14.13 | 34.47 | 6.77 |
| 71 | CAH 153 | 66.50 | 68.33 | 1.83 | 184.17 | 95.00 | 52.78 | 28.69 | 18.58 | 4.34 | 15.99 | 33.86 | 7.94 |
| 72 | CAH 1511 | 67.33 | 70.33 | 3.00 | 172.83 | 93.33 | 47.93 | 30.26 | 17.85 | 4.33 | 14.58 | 33.76 | 7.80 |
| 73 | 900 MG | 66.83 | 68.17 | 1.33 | 173.50 | 93.17 | 50.36 | 30.90 | 18.05 | 4.49 | 14.39 | 34.93 | 8.54 |
| 74 | P 3502 | 66.33 | 68.50 | 2.17 | 177.00 | 97.50 | 46.99 | 28.83 | 19.92 | 4.29 | 14.76 | 33.97 | 7.81 |
| 75 | Hytech5106 | 66.50 | 69.17 | 2.67 | 171.50 | 90.83 | 50.15 | 29.28 | 18.89 | 4.44 | 14.64 | 34.96 | 8.09 |
| Mean | | 67.18 | 69.60 | 2.42 | 170.96 | 90.66 | 48.30 | 29.05 | 18.26 | 4.34 | 14.80 | 33.73 | 7.87 |
| Max | | 70.83 | 73.83 | 4.17 | 196.67 | 117.50 | 61.08 | 32.62 | 20.57 | 5.06 | 18.33 | 38.60 | 10.48 |
| Min | | 62.67 | 64.67 | 1.33 | 142.67 | 73.33 | 42.19 | 24.99 | 16.16 | 3.88 | 13.65 | 27.58 | 5.47 |
| CV | | 1.97 | 2.16 | 33.29 | 6.08 | 10.49 | 6.00 | 2.95 | 4.26 | 3.78 | 3.39 | 4.13 | 5.89 |
| CD | | 1.92 | 2.12 | 0.59 | 11.65 | 10.02 | 3.76 | 1.80 | 0.99 | 0.25 | 0.91 | 2.37 | 1.14 |

D50A, days to 50% anthesis (#); D50S, days to 50% silking (#); ASI, anthesis-silking interval (#); PH, plant height (cm); EH, ear height (cm); SPAD, chlorophyll content (SPAD readings); TW, test weight (g); EL, ear length (cm); EG, ear girth (cm); KRE, number of kernel rows per ear (#); KNR, number of kernels per row (#);GY, grain yield (t/ha)

**Supplemental table 2b. Mean performance for recorded traits of 75 maize hybrids under managed drought stress**

| **Code** | **Genotype** | **D50A** | **D50S** | **ASI** | **PH** | **EH** | **SPAD** | **TW** | **EL** | **EG** | **KRE** | **KNR** | **GY** |
| --- | --- | --- | --- | --- | --- | --- | --- | --- | --- | --- | --- | --- | --- |
| 1 | ZH161271 | 88.75 | 95.50 | 6.75 | 145.00 | 95.00 | 37.59 | 23.98 | 16.42 | 4.24 | 14.67 | 25.82 | 3.87 |
| 2 | ZH161475 | 88.00 | 95.00 | 7.00 | 148.00 | 90.00 | 40.99 | 27.20 | 15.71 | 3.85 | 13.50 | 27.33 | 3.67 |
| 3 | ZH161493 | 91.25 | 96.25 | 5.00 | 157.50 | 108.75 | 39.13 | 26.25 | 17.92 | 4.24 | 15.00 | 33.58 | 4.37 |
| 4 | ZH161464 | 90.50 | 96.25 | 5.75 | 152.50 | 97.00 | 41.30 | 24.13 | 16.11 | 4.00 | 14.00 | 29.91 | 4.18 |
| 5 | ZH161409 | 89.50 | 96.25 | 6.75 | 145.00 | 102.50 | 39.62 | 23.60 | 16.10 | 3.55 | 12.42 | 27.87 | 2.97 |
| 6 | ZH161361 | 90.75 | 95.75 | 5.00 | 158.75 | 106.75 | 37.25 | 22.87 | 15.67 | 4.11 | 18.50 | 31.83 | 4.73 |
| 7 | ZH161485 | 91.25 | 96.50 | 5.25 | 145.00 | 87.00 | 41.13 | 25.98 | 16.34 | 3.72 | 14.42 | 30.64 | 3.73 |
| 8 | ZH161330 | 90.75 | 95.25 | 4.50 | 150.00 | 90.00 | 42.48 | 24.21 | 16.80 | 4.07 | 15.89 | 32.76 | 4.90 |
| 9 | ZH161384 | 90.75 | 95.25 | 4.50 | 153.75 | 101.75 | 41.22 | 21.87 | 18.54 | 3.88 | 16.28 | 37.23 | 5.90 |
| 10 | ZH161418 | 92.00 | 96.25 | 4.25 | 166.25 | 100.25 | 45.11 | 24.07 | 16.13 | 3.85 | 15.06 | 31.23 | 4.23 |
| 11 | ZH161276 | 90.50 | 96.00 | 5.50 | 146.25 | 101.25 | 36.26 | 23.20 | 15.24 | 3.86 | 14.47 | 31.42 | 3.73 |
| 12 | ZH161311 | 91.00 | 94.75 | 3.75 | 163.75 | 111.25 | 34.98 | 25.94 | 16.48 | 3.78 | 13.97 | 32.71 | 4.85 |
| 13 | ZH161358 | 90.00 | 96.25 | 6.25 | 159.25 | 94.00 | 38.13 | 24.38 | 15.98 | 3.87 | 13.96 | 33.01 | 4.59 |
| 14 | ZH161303 | 87.50 | 94.25 | 6.75 | 153.75 | 110.75 | 40.17 | 26.87 | 16.29 | 4.41 | 14.48 | 33.02 | 5.16 |
| 15 | ZH161438 | 91.50 | 94.75 | 3.25 | 179.00 | 111.75 | 40.26 | 26.74 | 16.92 | 4.08 | 14.90 | 33.17 | 5.31 |
| 16 | ZH161382 | 90.25 | 96.00 | 5.75 | 156.00 | 91.75 | 38.61 | 24.46 | 18.21 | 3.69 | 15.51 | 29.58 | 4.09 |
| 17 | ZH161434 | 89.00 | 95.75 | 6.75 | 166.25 | 93.25 | 39.65 | 25.91 | 16.65 | 3.90 | 16.44 | 32.23 | 4.51 |
| 18 | ZH161377 | 87.00 | 94.25 | 7.25 | 147.50 | 94.25 | 38.63 | 22.61 | 16.52 | 4.11 | 14.18 | 32.42 | 4.24 |
| 19 | ZH161458 | 89.00 | 92.75 | 3.75 | 155.50 | 98.00 | 43.11 | 24.88 | 19.20 | 4.16 | 14.55 | 37.58 | 5.44 |
| 20 | ZH161452 | 91.75 | 97.00 | 5.25 | 166.25 | 103.50 | 41.39 | 24.11 | 16.39 | 4.26 | 13.83 | 31.83 | 3.71 |
| 21 | ZH161473 | 90.50 | 95.50 | 5.00 | 154.75 | 104.50 | 41.08 | 23.52 | 16.73 | 3.88 | 14.33 | 32.50 | 4.30 |
| 22 | ZH161289 | 88.00 | 92.75 | 4.75 | 150.00 | 97.50 | 45.35 | 24.21 | 18.71 | 4.23 | 14.88 | 36.25 | 5.96 |
| 23 | ZH161471 | 89.00 | 94.50 | 5.50 | 147.50 | 99.50 | 35.03 | 23.31 | 14.05 | 3.74 | 14.47 | 30.42 | 3.44 |
| 24 | ZH161285 | 88.75 | 94.25 | 5.50 | 140.00 | 91.25 | 41.31 | 23.49 | 16.93 | 4.03 | 15.33 | 28.92 | 4.92 |
| 25 | ZH161135 | 88.00 | 94.75 | 6.75 | 147.50 | 81.25 | 38.40 | 27.55 | 16.04 | 4.23 | 14.47 | 31.58 | 4.53 |
| 26 | ZH161042 | 86.00 | 93.25 | 7.25 | 130.00 | 71.25 | 42.68 | 24.12 | 16.37 | 4.09 | 15.91 | 32.00 | 4.35 |
| 27 | ZH161079 | 89.75 | 96.50 | 6.75 | 151.50 | 85.50 | 40.17 | 25.49 | 16.58 | 3.84 | 13.65 | 32.58 | 3.82 |
| 28 | ZH161076 | 88.50 | 95.50 | 7.00 | 134.25 | 72.50 | 39.36 | 23.13 | 13.96 | 4.16 | 16.23 | 30.83 | 3.56 |
| 29 | ZH161039 | 86.75 | 95.75 | 9.00 | 140.00 | 80.00 | 44.19 | 27.21 | 15.98 | 4.05 | 14.83 | 31.83 | 4.42 |
| 30 | ZH161047 | 88.50 | 94.50 | 6.00 | 156.25 | 93.50 | 37.37 | 27.00 | 19.14 | 3.92 | 15.23 | 34.66 | 5.58 |
| 31 | ZH161100 | 88.50 | 93.75 | 5.25 | 140.00 | 81.50 | 39.68 | 25.18 | 16.94 | 3.73 | 16.50 | 31.33 | 4.55 |
| 32 | ZH161045 | 92.25 | 96.00 | 3.75 | 138.75 | 69.00 | 38.09 | 24.24 | 16.67 | 3.69 | 14.08 | 33.08 | 4.38 |
| 33 | ZH161095 | 86.75 | 94.50 | 7.75 | 143.75 | 85.25 | 33.19 | 26.48 | 16.98 | 3.73 | 14.17 | 27.16 | 2.60 |
| 34 | ZH161060 | 89.25 | 95.75 | 6.50 | 145.00 | 86.50 | 40.92 | 25.76 | 17.15 | 4.12 | 13.67 | 33.64 | 3.83 |
| 35 | ZH161054 | 91.50 | 95.50 | 4.00 | 161.00 | 92.75 | 38.05 | 26.50 | 16.88 | 4.25 | 14.57 | 32.33 | 3.80 |
| 36 | ZH161053 | 95.50 | 99.75 | 4.25 | 148.50 | 79.25 | 38.99 | 27.13 | 18.08 | 4.31 | 14.46 | 33.98 | 6.07 |
| 37 | ZH161083 | 86.00 | 93.75 | 7.75 | 150.00 | 85.75 | 41.22 | 26.16 | 17.83 | 4.37 | 16.13 | 31.50 | 4.42 |
| 38 | ZH161120 | 87.75 | 95.50 | 7.75 | 148.75 | 92.75 | 34.18 | 25.87 | 17.25 | 4.23 | 15.17 | 30.50 | 4.29 |
| 39 | ZH161038 | 90.25 | 94.50 | 4.25 | 158.75 | 93.75 | 39.45 | 24.04 | 18.06 | 3.53 | 14.32 | 33.50 | 4.15 |
| 40 | ZH161054 | 88.00 | 94.50 | 6.50 | 149.00 | 88.00 | 37.69 | 27.89 | 16.79 | 3.97 | 13.83 | 31.42 | 4.86 |
| 41 | ZH161051 | 88.00 | 93.25 | 5.25 | 148.75 | 94.00 | 40.86 | 27.43 | 18.21 | 4.20 | 15.60 | 36.33 | 5.81 |
| 42 | ZH161054 | 89.00 | 93.75 | 5.00 | 155.50 | 90.75 | 37.81 | 26.16 | 16.92 | 4.36 | 15.80 | 33.39 | 4.33 |
| 43 | ZH161082 | 85.75 | 95.25 | 9.50 | 150.50 | 85.75 | 35.09 | 27.00 | 15.71 | 3.85 | 13.50 | 27.67 | 2.93 |
| 44 | ZH161063 | 85.00 | 91.00 | 6.00 | 160.50 | 107.50 | 38.79 | 26.78 | 16.54 | 4.03 | 15.17 | 36.15 | 5.89 |
| 45 | ZH161093 | 88.00 | 94.75 | 6.75 | 133.75 | 70.00 | 34.82 | 25.17 | 15.81 | 4.38 | 14.50 | 32.08 | 4.04 |
| 46 | ZH161071 | 86.75 | 92.25 | 5.50 | 141.50 | 83.75 | 38.01 | 25.65 | 16.81 | 4.15 | 14.50 | 31.25 | 4.99 |
| 47 | ZH161210 | 89.75 | 95.25 | 5.50 | 156.25 | 92.75 | 40.71 | 22.66 | 15.73 | 4.84 | 18.23 | 29.50 | 4.04 |
| 48 | ZH161102 | 86.00 | 94.00 | 8.00 | 129.00 | 78.75 | 38.12 | 25.56 | 16.82 | 3.87 | 14.08 | 31.08 | 4.22 |
| 49 | ZH161398 | 90.50 | 95.75 | 5.25 | 167.50 | 103.75 | 43.00 | 24.43 | 16.39 | 3.84 | 13.83 | 32.35 | 5.60 |
| 50 | ZH161410 | 86.00 | 91.75 | 5.75 | 158.00 | 103.75 | 40.25 | 25.84 | 16.65 | 4.19 | 14.17 | 33.36 | 4.86 |
| 51 | ZH161478 | 91.75 | 96.75 | 5.00 | 139.25 | 86.75 | 33.28 | 24.36 | 16.12 | 4.42 | 14.45 | 31.18 | 4.80 |
| 52 | ZH161456 | 92.50 | 96.25 | 3.75 | 158.75 | 94.50 | 35.71 | 22.40 | 17.82 | 3.85 | 13.17 | 31.78 | 3.50 |
| 53 | ZH161484 | 89.50 | 93.25 | 3.75 | 166.25 | 107.25 | 37.90 | 25.14 | 16.73 | 4.03 | 15.83 | 35.42 | 4.42 |
| 54 | ZH161463 | 88.50 | 93.75 | 5.25 | 146.50 | 94.50 | 36.61 | 24.11 | 16.12 | 3.76 | 13.33 | 32.58 | 4.05 |
| 55 | ZH15449 | 89.00 | 94.75 | 5.75 | 153.00 | 81.75 | 39.68 | 25.02 | 16.86 | 4.07 | 14.83 | 34.00 | 4.24 |
| 56 | ZH161196 | 91.50 | 96.50 | 5.00 | 159.75 | 91.25 | 42.25 | 24.90 | 17.54 | 3.91 | 14.50 | 32.83 | 5.21 |
| 57 | ZH161194 | 92.00 | 96.50 | 4.50 | 139.00 | 70.25 | 34.21 | 25.04 | 16.38 | 3.79 | 14.00 | 28.83 | 3.06 |
| 58 | ZH161064 | 88.75 | 94.25 | 5.50 | 161.50 | 91.75 | 33.91 | 27.19 | 17.65 | 3.83 | 13.67 | 29.58 | 4.65 |
| 59 | ZH161068 | 89.50 | 95.00 | 5.50 | 140.25 | 88.50 | 36.02 | 26.71 | 17.28 | 3.83 | 15.83 | 31.92 | 4.65 |
| 60 | ZH161129 | 90.25 | 94.25 | 4.00 | 143.75 | 80.50 | 39.41 | 28.58 | 16.91 | 3.93 | 13.67 | 32.42 | 5.14 |
| 61 | ZH161077 | 90.00 | 95.00 | 5.25 | 169.25 | 100.50 | 46.45 | 27.65 | 18.48 | 3.91 | 14.33 | 33.77 | 5.05 |
| 62 | ZH161066 | 88.50 | 94.25 | 5.75 | 141.75 | 81.25 | 40.60 | 25.99 | 17.58 | 3.69 | 14.55 | 31.17 | 4.68 |
| 63 | ZH161137 | 87.00 | 96.00 | 9.00 | 141.25 | 80.00 | 38.23 | 26.98 | 15.40 | 4.05 | 14.40 | 30.38 | 3.12 |
| 64 | ZH161078 | 89.75 | 95.25 | 5.50 | 158.75 | 90.00 | 40.50 | 25.88 | 16.51 | 4.06 | 14.65 | 31.91 | 5.57 |
| 65 | ZH161114 | 90.25 | 97.00 | 6.75 | 176.75 | 98.75 | 39.34 | 24.58 | 15.30 | 3.72 | 14.25 | 27.92 | 2.58 |
| 66 | ZH161043 | 88.00 | 95.00 | 7.00 | 141.00 | 91.25 | 35.53 | 25.76 | 16.72 | 3.70 | 15.17 | 29.33 | 3.85 |
| 67 | ZH161184 | 88.25 | 95.00 | 6.75 | 139.75 | 82.00 | 36.56 | 26.43 | 17.53 | 3.93 | 13.46 | 31.33 | 3.89 |
| 68 | ZH161050 | 90.00 | 96.75 | 6.75 | 129.00 | 71.75 | 32.41 | 23.15 | 16.31 | 3.94 | 14.67 | 29.58 | 3.67 |
| 69 | ZH161207 | 89.50 | 95.25 | 5.75 | 144.50 | 84.50 | 41.80 | 26.05 | 16.65 | 3.61 | 14.09 | 25.42 | 3.02 |
| 70 | ZH161089 | 88.50 | 95.00 | 6.50 | 138.50 | 77.50 | 42.19 | 26.90 | 16.73 | 3.88 | 15.05 | 29.42 | 3.46 |
| 71 | CAH 153 | 90.50 | 94.50 | 4.00 | 168.75 | 87.50 | 36.41 | 29.35 | 17.08 | 3.77 | 14.33 | 29.92 | 4.50 |
| 72 | CAH 1511 | 90.50 | 94.50 | 4.00 | 164.00 | 82.50 | 39.09 | 29.80 | 17.48 | 4.23 | 15.54 | 34.78 | 5.07 |
| 73 | 900 MG | 89.75 | 93.50 | 3.75 | 160.25 | 100.50 | 39.02 | 23.53 | 16.10 | 4.14 | 15.67 | 34.33 | 4.77 |
| 74 | P 3502 | 88.75 | 94.00 | 5.25 | 166.00 | 104.25 | 46.93 | 22.38 | 18.94 | 4.05 | 15.00 | 35.71 | 5.03 |
| 75 | Hytech5106 | 89.75 | 95.00 | 5.25 | 153.00 | 93.50 | 40.55 | 26.36 | 16.72 | 3.80 | 14.06 | 32.75 | 4.57 |
| Mean | | 89.30 | 95.00 | 5.71 | 151.36 | 91.25 | 39.14 | 25.36 | 16.79 | 3.99 | 14.72 | 31.82 | 4.39 |
| Highest | | 95.50 | 99.75 | 9.50 | 179.00 | 111.75 | 46.93 | 29.80 | 19.20 | 4.84 | 18.50 | 37.58 | 6.07 |
| Lowest | | 85.00 | 91.00 | 3.25 | 129.00 | 69.00 | 32.41 | 21.87 | 13.96 | 3.53 | 12.42 | 25.42 | 2.58 |
| CV % | | 2.07 | 1.99 | 23.48 | 6.14 | 9.65 | 8.00 | 4.86 | 5.38 | 4.60 | 2.12 | 5.35 | 7.40 |
| CD | | 1.76 | 1.24 | 1.26 | 9.69 | 9.68 | 2.85 | 1.52 | 0.92 | 0.21 | 0.92 | 2.21 | 0.69 |

D50A, days to 50% anthesis (#); D50S, days to 50% silking (#); ASI, anthesis-silking interval (#); PH, plant height (cm); EH, ear height (cm); SPAD, chlorophyll content (SPAD readings); TW, test weight (g); EL, ear length (cm); EG, ear girth (cm); KRE, number of kernel rows per ear (#); KNR, number of kernels per row (#);GY, grain yield (t/ha)

**Supplemental Table 2c. Mean performance for recorded traits of 75 maize hybrids under managed waterlogging stress**

| **Code** | **Genotype** | **D50A** | **D50S** | **ASI** | **PH** | **EH** | **SPAD** | **TW** | **EL** | **EG** | **KRE** | **KNR** | **GY** |
| --- | --- | --- | --- | --- | --- | --- | --- | --- | --- | --- | --- | --- | --- |
| 1 | ZH161271 | 55.25 | 61.25 | 6.00 | 148.75 | 77.50 | 21.15 | 24.30 | 13.08 | 3.71 | 14.08 | 26.38 | 3.05 |
| 2 | ZH161475 | 57.00 | 61.50 | 4.50 | 118.75 | 62.50 | 16.72 | 21.35 | 11.91 | 3.58 | 12.08 | 22.71 | 2.03 |
| 3 | ZH161493 | 58.25 | 63.00 | 4.75 | 146.25 | 86.25 | 20.42 | 21.65 | 12.75 | 3.79 | 14.17 | 24.30 | 3.36 |
| 4 | ZH161464 | 59.00 | 64.00 | 5.00 | 147.50 | 81.25 | 28.38 | 24.95 | 15.24 | 4.06 | 14.66 | 31.00 | 4.41 |
| 5 | ZH161409 | 56.75 | 61.75 | 5.00 | 123.75 | 74.25 | 17.83 | 20.84 | 12.73 | 3.87 | 11.75 | 23.13 | 2.57 |
| 6 | ZH161361 | 56.50 | 63.50 | 7.00 | 140.50 | 77.50 | 31.48 | 21.03 | 11.95 | 4.88 | 18.92 | 24.36 | 3.45 |
| 7 | ZH161485 | 59.25 | 66.25 | 7.00 | 141.25 | 75.00 | 20.58 | 22.69 | 13.68 | 4.04 | 15.37 | 25.61 | 2.87 |
| 8 | ZH161330 | 57.50 | 60.00 | 2.50 | 137.50 | 85.00 | 36.93 | 26.50 | 14.75 | 4.13 | 14.49 | 27.75 | 5.00 |
| 9 | ZH161384 | 58.75 | 63.50 | 4.75 | 147.50 | 85.00 | 31.18 | 27.52 | 14.80 | 4.05 | 13.69 | 34.25 | 4.69 |
| 10 | ZH161418 | 59.25 | 65.75 | 6.50 | 146.25 | 73.75 | 24.83 | 24.56 | 13.33 | 3.88 | 13.98 | 25.63 | 3.80 |
| 11 | ZH161276 | 60.25 | 65.25 | 5.00 | 137.50 | 86.25 | 22.88 | 22.09 | 13.03 | 3.89 | 14.47 | 28.38 | 3.08 |
| 12 | ZH161311 | 55.25 | 64.25 | 9.00 | 141.25 | 82.50 | 17.54 | 21.61 | 11.98 | 3.72 | 12.48 | 23.83 | 2.63 |
| 13 | ZH161358 | 55.50 | 58.75 | 3.25 | 143.75 | 77.50 | 32.37 | 24.89 | 14.16 | 4.14 | 15.43 | 28.37 | 5.14 |
| 14 | ZH161303 | 55.75 | 58.00 | 2.25 | 155.00 | 83.75 | 29.73 | 25.22 | 13.66 | 4.37 | 15.96 | 27.50 | 5.90 |
| 15 | ZH161438 | 60.50 | 64.50 | 4.00 | 152.50 | 81.25 | 24.16 | 24.13 | 13.67 | 3.65 | 12.17 | 27.92 | 3.63 |
| 16 | ZH161382 | 57.50 | 62.00 | 4.50 | 138.75 | 80.00 | 20.02 | 23.09 | 12.85 | 3.71 | 15.67 | 22.90 | 2.70 |
| 17 | ZH161434 | 58.25 | 63.75 | 5.50 | 138.75 | 75.00 | 23.73 | 25.27 | 13.40 | 3.91 | 14.89 | 26.42 | 2.39 |
| 18 | ZH161377 | 55.50 | 57.50 | 2.00 | 143.75 | 87.50 | 23.78 | 24.24 | 13.44 | 3.82 | 12.17 | 26.33 | 3.20 |
| 19 | ZH161458 | 56.25 | 59.75 | 3.50 | 145.00 | 86.25 | 28.61 | 24.94 | 14.27 | 4.14 | 15.60 | 30.08 | 5.28 |
| 20 | ZH161452 | 57.00 | 61.50 | 4.50 | 146.25 | 88.75 | 20.20 | 23.63 | 12.94 | 4.38 | 12.25 | 25.03 | 2.67 |
| 21 | ZH161473 | 59.00 | 63.50 | 4.50 | 146.25 | 78.75 | 29.67 | 24.22 | 13.96 | 4.06 | 13.83 | 26.78 | 3.49 |
| 22 | ZH161289 | 56.50 | 59.50 | 3.00 | 153.75 | 83.75 | 32.57 | 25.53 | 15.21 | 4.06 | 14.88 | 32.87 | 5.94 |
| 23 | ZH161471 | 53.50 | 57.75 | 4.25 | 122.50 | 72.50 | 23.27 | 22.69 | 11.33 | 4.02 | 14.17 | 22.83 | 3.58 |
| 24 | ZH161285 | 60.50 | 65.50 | 5.00 | 133.75 | 68.75 | 24.71 | 22.48 | 12.50 | 3.98 | 14.29 | 24.41 | 2.45 |
| 25 | ZH161135 | 55.25 | 60.25 | 5.00 | 132.50 | 70.00 | 24.64 | 26.95 | 13.42 | 3.81 | 14.58 | 23.04 | 4.50 |
| 26 | ZH161042 | 55.00 | 59.00 | 4.00 | 138.75 | 75.00 | 26.30 | 23.44 | 15.30 | 3.77 | 14.73 | 26.36 | 4.08 |
| 27 | ZH161079 | 57.75 | 61.25 | 3.50 | 133.75 | 66.25 | 21.38 | 24.61 | 14.05 | 3.88 | 14.22 | 26.50 | 3.32 |
| 28 | ZH161076 | 52.25 | 57.75 | 5.50 | 122.50 | 62.50 | 27.45 | 27.03 | 14.59 | 4.09 | 16.33 | 26.41 | 4.08 |
| 29 | ZH161039 | 52.75 | 60.50 | 7.75 | 123.75 | 68.75 | 20.35 | 24.43 | 14.60 | 3.97 | 14.17 | 26.83 | 3.52 |
| 30 | ZH161047 | 54.50 | 58.75 | 4.25 | 140.00 | 82.50 | 32.35 | 27.41 | 16.94 | 4.31 | 14.76 | 31.74 | 6.21 |
| 31 | ZH161100 | 52.75 | 56.50 | 3.75 | 141.25 | 73.75 | 26.33 | 26.27 | 13.89 | 3.84 | 14.25 | 25.41 | 4.28 |
| 32 | ZH161045 | 57.75 | 61.25 | 3.50 | 143.75 | 82.50 | 24.37 | 25.20 | 13.97 | 3.81 | 13.14 | 22.65 | 3.83 |
| 33 | ZH161095 | 57.00 | 63.00 | 6.00 | 122.75 | 65.00 | 19.62 | 24.39 | 11.67 | 3.79 | 13.00 | 20.00 | 2.92 |
| 34 | ZH161060 | 56.25 | 61.75 | 5.50 | 128.75 | 72.50 | 25.35 | 24.08 | 14.50 | 3.39 | 13.30 | 24.46 | 3.85 |
| 35 | ZH161054 | 57.75 | 60.50 | 2.75 | 143.75 | 76.25 | 19.28 | 26.85 | 13.51 | 3.65 | 13.58 | 21.92 | 3.10 |
| 36 | ZH161053 | 49.75 | 58.00 | 8.25 | 132.50 | 78.75 | 29.41 | 27.71 | 14.03 | 3.79 | 15.52 | 25.12 | 5.27 |
| 37 | ZH161083 | 54.50 | 60.25 | 5.75 | 137.50 | 80.00 | 24.68 | 26.19 | 13.37 | 4.12 | 14.17 | 26.04 | 4.08 |
| 38 | ZH161120 | 56.75 | 62.50 | 5.75 | 129.25 | 67.50 | 23.58 | 24.48 | 14.42 | 3.52 | 13.90 | 24.08 | 3.48 |
| 39 | ZH161038 | 57.00 | 60.75 | 3.75 | 133.75 | 78.75 | 25.42 | 25.99 | 14.01 | 3.85 | 13.88 | 25.38 | 4.17 |
| 40 | ZH161054 | 56.00 | 60.75 | 4.75 | 132.50 | 75.00 | 19.15 | 26.17 | 13.35 | 3.95 | 13.31 | 21.88 | 2.70 |
| 41 | ZH161051 | 56.00 | 59.50 | 3.50 | 133.75 | 82.50 | 28.77 | 27.18 | 16.46 | 3.78 | 14.21 | 26.25 | 5.30 |
| 42 | ZH161054 | 57.00 | 62.00 | 5.00 | 135.00 | 73.75 | 23.32 | 25.35 | 13.60 | 4.21 | 14.58 | 22.13 | 3.43 |
| 43 | ZH161082 | 53.75 | 60.25 | 6.50 | 132.50 | 77.50 | 24.81 | 25.96 | 13.26 | 3.63 | 13.75 | 21.33 | 3.52 |
| 44 | ZH161063 | 56.25 | 60.75 | 4.50 | 118.75 | 72.50 | 30.60 | 27.53 | 15.62 | 3.99 | 15.13 | 27.21 | 5.57 |
| 45 | ZH161093 | 55.50 | 61.50 | 6.00 | 116.25 | 66.25 | 27.20 | 26.85 | 13.71 | 4.03 | 14.17 | 25.29 | 4.31 |
| 46 | ZH161071 | 53.00 | 57.75 | 4.75 | 123.75 | 66.25 | 22.65 | 24.71 | 13.11 | 3.80 | 13.13 | 25.01 | 3.73 |
| 47 | ZH161210 | 57.00 | 63.00 | 6.00 | 141.25 | 73.75 | 24.74 | 25.13 | 11.54 | 4.73 | 17.42 | 23.17 | 2.81 |
| 48 | ZH161102 | 53.50 | 57.50 | 4.00 | 125.00 | 57.50 | 24.16 | 26.44 | 11.73 | 3.60 | 13.65 | 23.32 | 3.51 |
| 49 | ZH161398 | 56.50 | 59.00 | 2.50 | 161.25 | 86.25 | 28.41 | 26.51 | 13.29 | 4.25 | 16.45 | 30.19 | 6.06 |
| 50 | ZH161410 | 55.00 | 57.75 | 2.75 | 148.75 | 83.75 | 28.46 | 23.42 | 14.13 | 4.06 | 14.42 | 26.76 | 3.53 |
| 51 | ZH161478 | 59.00 | 62.25 | 3.25 | 125.00 | 66.25 | 22.20 | 24.06 | 12.38 | 4.25 | 13.50 | 22.42 | 2.97 |
| 52 | ZH161456 | 59.25 | 65.00 | 5.75 | 146.25 | 72.50 | 19.30 | 22.62 | 12.67 | 3.49 | 13.42 | 25.28 | 1.76 |
| 53 | ZH161484 | 58.25 | 65.50 | 7.25 | 153.75 | 90.00 | 23.36 | 24.08 | 15.29 | 3.88 | 15.00 | 24.67 | 2.91 |
| 54 | ZH161463 | 55.75 | 63.75 | 8.00 | 140.00 | 68.75 | 21.68 | 22.91 | 12.85 | 3.97 | 14.17 | 24.74 | 2.58 |
| 55 | ZH15449 | 57.25 | 61.00 | 3.75 | 147.50 | 81.25 | 27.52 | 26.82 | 14.08 | 3.88 | 14.54 | 33.09 | 4.76 |
| 56 | ZH161196 | 57.50 | 62.50 | 5.00 | 146.25 | 82.50 | 25.01 | 25.20 | 13.93 | 3.81 | 13.92 | 25.90 | 3.62 |
| 57 | ZH161194 | 60.00 | 66.25 | 6.25 | 132.75 | 67.50 | 18.19 | 23.98 | 13.31 | 3.68 | 13.83 | 21.92 | 2.97 |
| 58 | ZH161064 | 54.75 | 58.75 | 4.00 | 132.50 | 72.50 | 25.95 | 28.11 | 14.98 | 3.95 | 15.26 | 27.50 | 4.90 |
| 59 | ZH161068 | 52.00 | 55.75 | 3.75 | 136.25 | 72.50 | 25.05 | 29.16 | 17.51 | 4.21 | 14.19 | 32.17 | 4.50 |
| 60 | ZH161129 | 54.25 | 59.00 | 4.75 | 135.00 | 78.75 | 29.94 | 28.11 | 13.54 | 3.77 | 14.96 | 25.71 | 5.17 |
| 61 | ZH161077 | 55.75 | 59.50 | 3.75 | 141.25 | 66.25 | 28.69 | 28.51 | 13.46 | 3.89 | 14.48 | 25.65 | 4.92 |
| 62 | ZH161066 | 56.75 | 62.50 | 5.75 | 123.75 | 66.25 | 21.80 | 24.82 | 14.04 | 3.70 | 14.58 | 25.04 | 3.05 |
| 63 | ZH161137 | 57.50 | 62.75 | 5.25 | 142.50 | 78.75 | 25.30 | 26.45 | 15.93 | 3.55 | 12.89 | 27.98 | 2.57 |
| 64 | ZH161078 | 56.25 | 62.25 | 6.00 | 141.25 | 80.00 | 30.55 | 27.02 | 14.99 | 4.11 | 14.58 | 26.26 | 4.57 |
| 65 | ZH161114 | 58.00 | 63.00 | 5.00 | 153.75 | 78.75 | 21.58 | 24.77 | 13.06 | 3.84 | 13.54 | 23.05 | 3.15 |
| 66 | ZH161043 | 52.75 | 61.00 | 8.25 | 130.00 | 76.25 | 23.35 | 28.34 | 14.46 | 3.89 | 14.00 | 26.56 | 4.30 |
| 67 | ZH161184 | 54.25 | 57.75 | 3.50 | 127.50 | 68.75 | 22.73 | 24.61 | 13.92 | 3.78 | 14.58 | 22.86 | 3.37 |
| 68 | ZH161050 | 58.75 | 65.00 | 6.25 | 145.00 | 81.25 | 22.75 | 25.33 | 15.58 | 3.75 | 14.55 | 23.79 | 3.28 |
| 69 | ZH161207 | 55.75 | 62.00 | 6.25 | 135.00 | 68.75 | 23.61 | 27.06 | 15.41 | 3.80 | 13.83 | 23.58 | 4.06 |
| 70 | ZH161089 | 55.75 | 62.00 | 6.25 | 131.25 | 66.25 | 28.89 | 24.14 | 15.88 | 3.55 | 13.33 | 24.50 | 3.44 |
| 71 | CAH 153 | 57.25 | 61.75 | 4.50 | 143.75 | 85.00 | 25.49 | 23.07 | 14.92 | 4.00 | 14.58 | 30.54 | 3.68 |
| 72 | CAH 1511 | 55.75 | 58.75 | 3.00 | 150.00 | 90.00 | 26.99 | 24.60 | 14.70 | 4.22 | 13.88 | 25.02 | 3.89 |
| 73 | 900 MG | 57.00 | 59.75 | 2.75 | 158.75 | 83.75 | 22.53 | 27.70 | 16.08 | 4.07 | 13.80 | 28.85 | 4.07 |
| 74 | P 3502 | 57.25 | 62.00 | 4.50 | 151.25 | 87.50 | 31.53 | 25.57 | 14.29 | 3.98 | 13.15 | 27.21 | 3.72 |
| 75 | Hytech5106 | 53.00 | 58.50 | 5.25 | 146.75 | 86.75 | 28.62 | 26.03 | 13.77 | 4.19 | 13.17 | 26.60 | 4.04 |
| Mean | | 89.30 | 56.33 | 61.23 | 4.89 | 138.49 | 76.56 | 25.04 | 25.10 | 13.92 | 3.93 | 14.23 | 27.14 |
| Highest | | 95.50 | 60.50 | 66.25 | 9.00 | 161.25 | 90.00 | 36.93 | 29.16 | 17.51 | 4.88 | 18.92 | 54.08 |
| Lowest | | 85.00 | 49.75 | 55.75 | 2.00 | 116.25 | 57.50 | 16.72 | 20.84 | 11.33 | 3.39 | 11.75 | 8.07 |
| CV % | | 2.07 | 3.58 | 3.64 | 22.22 | 9.39 | 13.35 | 9.85 | 4.10 | 3.76 | 3.88 | 3.44 | 18.41 |
| CD | | 1.76 | 1.93 | 2.24 | 1.40 | 8.40 | 6.79 | 3.76 | 1.67 | 1.09 | 0.23 | 9.05 | 9.06 |

D50A, days to 50% anthesis (#); D50S, days to 50% silking (#); ASI, anthesis-silking interval (#); PH, plant height (cm); EH, ear height (cm); SPAD, chlorophyll content (SPAD readings); TW, test weight (g); EL, ear length (cm); EG, ear girth (cm); KRE, number of kernel rows per ear (#); KNR, number of kernels per row (#); GY, grain yield (t/ha)

**Supplemental Table 3. The proportions of total variation explained by genotype, environment and their interactions (GEI) under individual moisture regime**

| Sl.No. | Traits | Source | Optimal | | Drought | | Waterlogging | |
| --- | --- | --- | --- | --- | --- | --- | --- | --- |
|  |  |  | Variance | Percent | Variance | Percent | Variance | Percent |
| 1 | D50A | Block (Rep:Env) | 0.30 | 6.44 | 0.20 | 2.83 | 0.46 | 5.01 |
|  |  | Genotype | 2.42 | 52.75 | 1.88 | 26.49 | 2.25 | 24.54 |
|  |  | GEI | 0.53 | 11.59 | 1.45 | 20.48 | 2.86 | 31.20 |
|  |  | Residual | 1.34 | 29.21 | 3.56 | 50.20 | 3.59 | 39.25 |
| 2 | D50S | Block (Rep:Env) | 0.66 | 11.31 | 0.61 | 11.82 | 0.62 | 4.63 |
|  |  | Genotype | 2.79 | 48.10 | 0.48 | 9.45 | 1.59 | 11.91 |
|  |  | GEI | 0.85 | 14.59 | 0.69 | 13.46 | 6.87 | 51.52 |
|  |  | Residual | 1.51 | 26.00 | 3.34 | 65.27 | 4.26 | 31.95 |
| 3 | ASI | Block (Rep:Env) | 0.04 | 3.93 | 0.00 | 0.00 | 0.00 | 0.00 |
|  |  | Genotype | 0.02 | 1.81 | 0.69 | 18.03 | 0.40 | 8.35 |
|  |  | GEI | 0.40 | 37.24 | 1.37 | 35.76 | 3.18 | 66.44 |
|  |  | Residual | 0.62 | 57.01 | 1.77 | 46.21 | 1.20 | 25.20 |
| 4 | PH | Block (Rep:Env) | 22.18 | 7.46 | 25.14 | 12.87 | 50.41 | 21.64 |
|  |  | Genotype | 44.71 | 15.03 | 84.97 | 43.51 | 45.32 | 19.45 |
|  |  | GEI | 161.60 | 54.34 | 10.45 | 5.35 | 14.22 | 6.11 |
|  |  | Residual | 68.91 | 23.17 | 74.73 | 38.26 | 123.00 | 52.80 |
| 5 | EH | Block (Rep:Env) | 6.90 | 4.08 | 9.38 | 5.03 | 23.95 | 16.10 |
|  |  | Genotype | 61.91 | 36.64 | 73.79 | 39.62 | 20.53 | 13.80 |
|  |  | GEI | 29.82 | 17.65 | 33.86 | 18.18 | 11.83 | 7.95 |
|  |  | Residual | 70.33 | 41.63 | 69.20 | 37.16 | 92.44 | 62.15 |
| 6 | SPAD | Block (Rep:Env) | 0.41 | 1.23 | 0.73 | 3.81 | 0.00 | 0.00 |
|  |  | Genotype | 1.91 | 5.68 | 4.79 | 24.98 | 10.99 | 41.12 |
|  |  | GEI | 23.85 | 71.04 | 4.31 | 22.47 | 9.87 | 36.93 |
|  |  | Residual | 7.40 | 22.05 | 9.34 | 48.74 | 5.87 | 21.96 |
| 7 | TW | Block (Rep:Env) | 0.00 | 0.00 | 0.17 | 3.38 | 0.24 | 3.71 |
|  |  | Genotype | 1.03 | 17.51 | 1.42 | 28.08 | 1.46 | 23.02 |
|  |  | GEI | 4.14 | 70.26 | 2.26 | 44.66 | 3.66 | 57.75 |
|  |  | Residual | 0.72 | 12.23 | 1.21 | 23.88 | 0.98 | 15.52 |
| 8 | EL | Block (Rep:Env) | 0.07 | 3.64 | 0.01 | 0.00 | 0.00 | 0.00 |
|  |  | Genotype | 0.33 | 16.26 | 2.61 | 20.12 | 0.89 | 37.40 |
|  |  | GEI | 1.21 | 59.37 | 5.22 | 45.79 | 1.23 | 51.97 |
|  |  | Residual | 0.42 | 20.73 | 3.15 | 34.09 | 0.25 | 10.63 |
| 9 | EG | Block (Rep:Env) | 0.00 | 1.51 | 0.00 | 1.83 | 0.00 | 1.10 |
|  |  | Genotype | 0.02 | 17.69 | 0.02 | 14.45 | 0.04 | 33.96 |
|  |  | GEI | 0.07 | 59.29 | 0.06 | 57.33 | 0.05 | 44.66 |
|  |  | Residual | 0.03 | 21.52 | 0.03 | 26.38 | 0.02 | 20.29 |
| 10 | KRE | Block (Rep:Env) | 0.01 | 0.61 | 0.00 | 0.00 | 0.00 | 0.00 |
|  |  | Genotype | 0.35 | 25.09 | 0.65 | 38.30 | 0.61 | 27.57 |
|  |  | GEI | 0.81 | 57.96 | 0.59 | 35.29 | 1.36 | 61.59 |
|  |  | Residual | 0.23 | 16.34 | 0.45 | 26.41 | 0.24 | 10.84 |
| 11 | KNR | Block (Rep:Env) | 0.47 | 3.69 | 0.01 | 0.11 | 0.00 | 0.00 |
|  |  | Genotype | 1.00 | 7.86 | 2.58 | 22.55 | 4.50 | 34.48 |
|  |  | GEI | 9.64 | 76.08 | 5.05 | 44.25 | 7.28 | 55.71 |
|  |  | Residual | 1.57 | 12.36 | 3.78 | 33.09 | 1.28 | 9.81 |
| 12 | GY | Block (Rep:Env) | 0.05 | 2.64 | 0.01 | 0.63 | 0.01 | 0.72 |
|  |  | Genotype | 0.59 | 28.35 | 0.38 | 39.05 | 0.56 | 38.75 |
|  |  | GEI | 1.27 | 60.76 | 0.48 | 50.04 | 0.79 | 54.36 |
|  |  | Residual | 0.17 | 8.25 | 0.10 | 10.28 | 0.09 | 6.17 |

D50A, days to 50% anthesis (#); D50S, days to 50% silking (#); ASI, anthesis-silking interval (#); PH, plant height (cm); EH, ear height (cm); SPAD, chlorophyll content (SPAD readings); TW, test weight (g); EL, ear length (cm); EG, ear girth (cm); KRE, number of kernel rows per ear (#); KNR, number of kernels per row (#); GY, grain yield (t/ha)

**Supplemental Table 4. Correlation among 12 agronomic characters for 75 maize hybrids evaluated under optimal, drought and waterlogging.**

| **Traits** | **D50A** | **D50S** | **ASI** | **PH** | **EH** | **SPAD** | **TW** | **EL** | **EG** | **KRE** | **KNR** | **GY** |
| --- | --- | --- | --- | --- | --- | --- | --- | --- | --- | --- | --- | --- |
|  | **Optimal** | | | | | | | | | | | |
| **D50A** | 1.00 |  |  |  |  |  |  |  |  |  |  |  |
| **D50S** | 0.96 | 1.00 |  |  |  |  |  |  |  |  |  |  |
| **ASI** | 0.23 | 0.47 | 1.00 |  |  |  |  |  |  |  |  |  |
| **PH** | 0.11 | 0.10 | -0.01 | 1.00 |  |  |  |  |  |  |  |  |
| **EH** | 0.25 | 0.24 | 0.04 | 0.78 | 1.00 |  |  |  |  |  |  |  |
| **SPAD** | -0.12 | -0.11 | -0.01 | 0.48 | 0.38 | 1.00 |  |  |  |  |  |  |
| **TW** | -0.29 | -0.30 | -0.13 | 0.21 | -0.11 | 0.39 | 1.00 |  |  |  |  |  |
| **EL** | -0.07 | -0.09 | -0.10 | 0.43 | 0.28 | 0.44 | 0.37 | 1.00 |  |  |  |  |
| **EG** | -0.05 | -0.05 | 0.00 | 0.31 | 0.28 | 0.42 | 0.22 | 0.17 | 1.00 |  |  |  |
| **KRE** | -0.06 | -0.01 | 0.17 | 0.46 | 0.36 | 0.44 | 0.23 | 0.30 | 0.68 | 1.00 |  |  |
| **KNR** | 0.03 | 0.02 | -0.04 | 0.57 | 0.57 | 0.54 | 0.20 | 0.63 | 0.39 | 0.41 | 1.00 |  |
| **GY** | -0.20 | -0.20 | -0.06 | 0.63 | 0.42 | 0.69 | 0.53 | 0.52 | 0.53 | 0.61 | 0.74 | 1.00 |
| **Managed Drought** | | | | | | | | | | | | |
| **D50A** | 1.00 |  |  |  |  |  |  |  |  |  |  |  |
| **D50S** | 0.69 | 1.00 |  |  |  |  |  |  |  |  |  |  |
| **ASI** | -0.70 | 0.03 | 1.00 |  |  |  |  |  |  |  |  |  |
| **PH** | 0.33 | 0.00 | -0.45 | 1.00 |  |  |  |  |  |  |  |  |
| **EH** | 0.13 | -0.16 | -0.33 | 0.72 | 1.00 |  |  |  |  |  |  |  |
| **SPAD** | 0.01 | -0.11 | -0.12 | 0.27 | 0.21 | 1.00 |  |  |  |  |  |  |
| **TW** | -0.18 | -0.15 | 0.11 | 0.08 | -0.20 | -0.07 | 1.00 |  |  |  |  |  |
| **EL** | 0.10 | -0.19 | -0.32 | 0.21 | 0.12 | 0.28 | 0.18 | 1.00 |  |  |  |  |
| **EG** | -0.09 | -0.11 | 0.02 | 0.02 | 0.04 | 0.09 | 0.01 | 0.00 | 1.00 |  |  |  |
| **KRE** | 0.09 | -0.33 | -0.45 | 0.31 | 0.30 | 0.34 | 0.00 | 0.49 | 0.28 | 1.00 |  |  |
| **KNR** | -0.05 | -0.16 | -0.10 | 0.06 | 0.05 | 0.14 | -0.19 | 0.02 | 0.42 | 0.21 | 1.00 |  |
| **GY** | -0.08 | -0.33 | -0.43 | 0.27 | 0.28 | 0.34 | 0.15 | 0.52 | 0.32 | 0.73 | 0.27 | 1.00 |
| **Managed Waterlogging** | | | | | | | | | | | | |
| **D50A** | 1.00 |  |  |  |  |  |  |  |  |  |  |  |
| **D50S** | 0.80 | 1.00 |  |  |  |  |  |  |  |  |  |  |
| **ASI** | -0.11 | 0.51 | 1.00 |  |  |  |  |  |  |  |  |  |
| **PH** | 0.33 | 0.13 | -0.26 | 1.00 |  |  |  |  |  |  |  |  |
| **EH** | 0.18 | 0.02 | -0.23 | 0.75 | 1.00 |  |  |  |  |  |  |  |
| **SPAD** | -0.18 | -0.34 | -0.30 | 0.24 | 0.32 | 1.00 |  |  |  |  |  |  |
| **TW** | -0.41 | -0.46 | -0.18 | 0.01 | -0.01 | 0.45 | 1.00 |  |  |  |  |  |
| **EL** | -0.14 | -0.21 | -0.16 | 0.17 | 0.24 | 0.45 | 0.54 | 1.00 |  |  |  |  |
| **EG** | -0.06 | -0.14 | -0.15 | 0.24 | 0.29 | 0.41 | 0.05 | -0.03 | 1.00 |  |  |  |
| **KRE** | -0.12 | -0.08 | 0.04 | 0.12 | 0.04 | 0.44 | 0.14 | 0.05 | 0.57 | 1.00 |  |  |
| **KNR** | 0.00 | -0.18 | -0.29 | 0.43 | 0.43 | 0.57 | 0.33 | 0.56 | 0.28 | 0.20 | 1.00 |  |
| **GY** | -0.37 | -0.53 | -0.35 | 0.17 | 0.26 | 0.76 | 0.64 | 0.50 | 0.30 | 0.39 | 0.58 | 1.00 |

D50A, days to 50% anthesis (#); D50S, days to 50% silking (#); ASI, anthesis-silking interval (#); PH, plant height (cm); EH, ear height (cm); SPAD, chlorophyll content (SPAD readings); TW, test weight (g); EL, ear length (cm); EG, ear girth (cm); KRE, number of kernel rows per ear (#); KNR, number of kernels per row (#); GY, grain yield (t/ha)

**Supplemental Table 5. List of the maize hybrids selected in the individual moisture condition and across the moisture regimes**

| **Optimal** | | **Managed Drought** | | **Managed Waterlogging** | | **Hybrids in common across moisture regimes** | |
| --- | --- | --- | --- | --- | --- | --- | --- |
| **Genotype Code** | **Name of genotype** | **Genotype Code** | **Name of genotype** | **Genotype Code** | **Name of genotype** | **Genotype Code** | **Name of genotype** |
| 14 | ZH161303 | 22 | ZH161289 | 49 | ZH161398 | 22 | ZH161289 |
| 26 | ZH161042 | 44 | ZH161063 | 14 | ZH161303 | 14 | ZH161303 |
| 30 | ZH161047 | 19 | ZH161458 | 30 | ZH161047 |  |  |
| 22 | ZH161289 | 41 | ZH161051 | 13 | ZH161358 |  |  |
| 60 | ZH161129 | 74 | P3502 | 19 | ZH161458 |  |  |
| 58 | ZH161064 | 50 | ZH161410 | 22 | ZH161289 |  |  |
| 44 | ZH161063 | 14 | ZH161303 | 8 | ZH161330 |  |  |
| 49 | ZH161398 | 37 | ZH161083 | 60 | ZH161129 |  |  |
| 64 | ZH161078 | 9 | ZH161384 | 75 | Hytech5106 |  |  |
| 59 | ZH161068 | 53 | ZH161484 | 58 | ZH161064 |  |  |
| 36 | ZH161053 | 46 | ZH161071 | 64 | ZH161078 |  |  |

**Supplemental Table 6. Multi-trait genotype-ideotype distance index (MGIDI) values and final loadings of factors (FAs) contributing to the MGIDI values for 75 maize hybrids under tested moisture regimes**

| **Code** | **Genotype Name** | **Optimal** | | | | **Managed Drought** | | | | | **Managed Waterlogging** | | | | |
| --- | --- | --- | --- | --- | --- | --- | --- | --- | --- | --- | --- | --- | --- | --- | --- |
|  |  | **FA1** | **FA2** | **FA3** | **MGIDI** | **FA1** | **FA2** | **FA3** | **FA4** | **MGIDI** | **FA1** | **FA2** | **FA3** | **FA4** | **MGIDI** |
| 1 | ZH161271 | -2.96 | -2.63 | 0.61 | 4.83 | -1.88 | 2.92 | -1.57 | -2.10 | 5.58 | -1.49 | -1.37 | -1.41 | 2.15 | 5.33 |
| 2 | ZH161475 | -3.48 | -3.47 | 0.41 | 4.12 | -2.46 | 3.46 | 0.15 | -1.84 | 5.92 | 0.05 | -0.23 | -0.15 | 2.96 | 7.23 |
| 3 | ZH161493 | -3.94 | -1.90 | -0.15 | 5.21 | -3.92 | 1.92 | -1.33 | -2.57 | 4.66 | -0.56 | -2.07 | -1.54 | 2.30 | 5.71 |
| 4 | ZH161464 | -3.55 | -2.09 | -0.78 | 5.62 | -2.54 | 2.19 | -1.01 | -2.66 | 5.51 | -2.91 | -2.53 | -2.02 | 1.39 | 4.53 |
| 5 | ZH161409 | -3.76 | -2.52 | -0.37 | 5.00 | -1.46 | 2.58 | 0.70 | -2.94 | 7.01 | -0.14 | -0.97 | -0.58 | 2.91 | 6.64 |
| 6 | ZH161361 | -4.72 | -2.11 | 3.16 | 3.81 | -2.24 | 2.05 | -3.81 | -3.22 | 5.10 | -0.23 | -1.53 | -6.47 | 1.15 | 5.13 |
| 7 | ZH161485 | -3.13 | -1.72 | 0.19 | 5.54 | -3.01 | 1.63 | -0.45 | -1.57 | 6.08 | -1.23 | -1.99 | -2.38 | 0.36 | 5.66 |
| 8 | ZH161330 | -4.41 | -2.64 | 0.62 | 4.06 | -3.66 | 1.93 | -2.33 | -1.99 | 4.59 | -2.85 | -2.03 | -2.38 | 3.37 | **3.48** |
| 9 | ZH161384 | -4.58 | -1.20 | -1.09 | 6.09 | -4.49 | 1.92 | -2.50 | -3.02 | **3.82** | -3.60 | -2.72 | -1.61 | 1.63 | 4.58 |
| 10 | ZH161418 | -4.48 | -1.41 | 0.00 | 5.31 | -2.83 | 1.43 | -1.32 | -3.47 | 5.53 | -1.86 | -2.01 | -1.66 | 0.77 | 5.53 |
| 11 | ZH161276 | -3.06 | -1.69 | -1.30 | 6.43 | -1.81 | 2.03 | -1.36 | -2.80 | 5.96 | -1.07 | -2.96 | -1.78 | 1.68 | 5.50 |
| 12 | ZH161311 | -4.11 | -2.55 | -0.72 | 5.07 | -3.53 | 2.40 | -0.10 | -3.54 | 5.21 | -0.66 | -1.41 | -1.14 | 1.04 | 6.42 |
| 13 | ZH161358 | -2.79 | -2.20 | 0.12 | 5.44 | -2.88 | 2.38 | -0.83 | -2.60 | 5.28 | -2.31 | -1.71 | -2.94 | 3.43 | **3.33** |
| 14 | ZH161303 | -5.31 | -3.15 | 1.51 | **2.91¶** | -3.52 | 3.99 | -1.67 | -2.86 | **3.68** | -1.95 | -1.98 | -3.52 | 4.01 | **3.13** |
| 15 | ZH161438 | -3.43 | -0.65 | 0.13 | 6.24 | -4.15 | 2.09 | -0.96 | -3.96 | 4.53 | -1.82 | -3.03 | -0.12 | 2.16 | 6.24 |
| 16 | ZH161382 | -2.56 | -2.57 | 0.66 | 5.11 | -3.26 | 2.34 | -0.69 | -2.21 | 5.22 | -0.58 | -1.43 | -1.93 | 2.26 | 5.49 |
| 17 | ZH161434 | -3.04 | -1.87 | 0.24 | 5.46 | -3.13 | 3.01 | -1.62 | -2.70 | 4.40 | -1.52 | -1.54 | -1.88 | 1.39 | 5.26 |
| 18 | ZH161377 | -3.48 | -4.16 | -1.22 | 4.98 | -2.53 | 3.85 | -1.69 | -2.49 | 4.60 | -1.20 | -2.03 | -0.50 | 4.77 | 5.97 |
| 19 | ZH161458 | -4.59 | -2.98 | 0.43 | 3.85 | -5.54 | 3.17 | -1.47 | -2.74 | **3.06** | -2.44 | -2.20 | -2.82 | 3.03 | **3.39** |
| 20 | ZH161452 | -3.78 | -2.22 | -0.51 | 5.27 | -2.59 | 1.52 | -1.27 | -3.33 | 5.66 | -0.49 | -2.59 | -1.64 | 3.29 | 5.51 |
| 21 | ZH161473 | -3.17 | -1.54 | 0.36 | 5.58 | -2.95 | 2.30 | -1.05 | -3.36 | 5.07 | -1.83 | -2.47 | -1.85 | 1.92 | 4.86 |
| 22 | ZH161289 | -5.58 | -2.73 | 1.44 | **3.25** | -5.23 | 3.71 | -2.13 | -2.59 | **2.49** | -3.39 | -2.66 | -2.29 | 3.23 | **3.43** |
| 23 | ZH161471 | -1.98 | -2.80 | -0.17 | 5.77 | -1.22 | 2.87 | -1.15 | -2.92 | 6.14 | -0.18 | -0.13 | -2.46 | 4.06 | 5.54 |
| 24 | ZH161285 | -1.82 | -1.29 | -0.48 | 6.89 | -3.06 | 2.81 | -2.02 | -1.81 | 4.65 | -0.57 | -1.70 | -1.94 | 1.38 | 5.79 |
| 25 | ZH161135 | -2.86 | -3.70 | -0.78 | 5.16 | -3.52 | 3.44 | -1.28 | -0.94 | 4.82 | -2.03 | -0.34 | -1.84 | 2.52 | 4.87 |
| 26 | ZH161042 | -4.03 | -4.76 | 0.77 | **2.97** | -3.13 | 4.15 | -2.57 | -0.74 | 4.54 | -2.25 | -1.13 | -1.57 | 2.87 | 4.66 |
| 27 | ZH161079 | -1.97 | -1.76 | 1.50 | 5.78 | -3.09 | 2.46 | -0.37 | -1.75 | 5.59 | -1.55 | -1.03 | -1.28 | 2.61 | 5.33 |
| 28 | ZH161076 | -1.28 | -3.21 | 0.34 | 5.88 | -1.53 | 2.72 | -3.24 | -0.78 | 6.08 | -2.62 | 0.97 | -3.12 | 2.40 | 4.50 |
| 29 | ZH161039 | -2.16 | -3.51 | -0.39 | 5.45 | -3.15 | 3.89 | -1.39 | -0.83 | 4.94 | -2.22 | 0.35 | -1.89 | 1.38 | 5.46 |
| 30 | ZH161047 | -3.96 | -3.43 | 2.73 | **3.04** | -5.11 | 3.09 | -0.79 | -1.71 | 4.10 | -4.21 | -1.46 | -2.55 | 2.68 | **3.22** |
| 31 | ZH161100 | -2.69 | -3.46 | -0.39 | 5.10 | -3.58 | 3.24 | -1.74 | -1.42 | 4.39 | -2.09 | -0.45 | -1.76 | 3.95 | 4.65 |
| 32 | ZH161045 | -1.40 | -1.55 | -0.78 | 7.15 | -3.84 | 0.76 | -0.77 | -0.46 | 6.50 | -1.55 | -1.90 | -0.89 | 3.01 | 5.46 |
| 33 | ZH161095 | -1.72 | -2.92 | 0.98 | 5.42 | -2.10 | 3.83 | 0.29 | -1.21 | 6.38 | -0.43 | 0.07 | -1.19 | 1.94 | 6.52 |
| 34 | ZH161060 | -2.67 | -3.73 | -0.73 | 5.23 | -3.57 | 2.75 | -0.90 | -1.42 | 5.01 | -2.33 | -0.75 | -0.41 | 1.91 | 5.96 |
| 35 | ZH161054 | -4.35 | -3.21 | -1.51 | 5.22 | -3.60 | 1.68 | -1.19 | -2.03 | 5.20 | -1.28 | -1.45 | -0.50 | 3.35 | 5.92 |
| 36 | ZH161053 | -4.31 | -3.92 | -0.11 | **3.79** | -5.28 | -0.68 | -1.52 | -0.16 | 7.04 | -3.31 | 0.68 | -2.87 | 1.86 | 4.45 |
| 37 | ZH161083 | -1.90 | -4.03 | 0.13 | 5.17 | -3.73 | 4.46 | -2.27 | -1.45 | **3.72** | -1.94 | -0.72 | -2.31 | 2.48 | 4.46 |
| 38 | ZH161120 | -1.62 | -2.53 | 0.87 | 5.73 | -2.94 | 3.27 | -1.60 | -1.34 | 4.91 | -2.11 | -0.45 | -0.80 | 1.57 | 5.92 |
| 39 | ZH161038 | -3.68 | -2.99 | -0.25 | 4.68 | -3.82 | 2.29 | -0.08 | -2.71 | 5.21 | -2.03 | -1.27 | -1.37 | 2.90 | 4.90 |
| 40 | ZH161054 | -1.97 | -3.73 | 0.64 | 4.98 | -3.86 | 3.49 | -0.26 | -1.47 | 4.95 | -1.12 | -0.79 | -1.20 | 2.88 | 5.62 |
| 41 | ZH161051 | -4.19 | -3.11 | 0.50 | 3.88 | -5.37 | 3.67 | -1.76 | -1.72 | **3.13** | -3.50 | -1.27 | -1.16 | 2.86 | 4.59 |
| 42 | ZH161054 | -3.42 | -3.66 | -0.50 | 4.65 | -3.77 | 2.87 | -2.28 | -1.70 | 4.09 | -1.24 | -0.81 | -2.39 | 2.08 | 4.94 |
| 43 | ZH161082 | -2.38 | -4.46 | 0.17 | 4.64 | -1.69 | 4.44 | 0.25 | -1.53 | 6.45 | -1.85 | -0.12 | -1.34 | 2.13 | 5.52 |
| 44 | ZH161063 | -3.21 | -4.30 | 1.06 | **3.57** | -4.35 | 5.30 | -1.49 | -3.33 | **2.98** | -3.53 | -0.10 | -2.19 | 1.96 | 4.37 |
| 45 | ZH161093 | -3.18 | -2.89 | -0.24 | 4.99 | -2.99 | 2.98 | -2.23 | 0.19 | 5.65 | -2.37 | 0.40 | -2.14 | 1.72 | 5.11 |
| 46 | ZH161071 | -2.66 | -3.97 | -0.40 | 4.94 | -3.84 | 4.25 | -1.47 | -1.50 | **4.01** | -1.54 | 0.11 | -1.25 | 3.54 | 5.56 |
| 47 | ZH161210 | -2.37 | -2.59 | 3.18 | 4.79 | -2.02 | 2.24 | -5.09 | -2.18 | 5.56 | -0.26 | -1.04 | -5.02 | 1.60 | 4.87 |
| 48 | ZH161102 | -3.09 | -5.24 | -0.01 | 4.07 | -3.19 | 4.45 | -0.80 | -0.62 | 5.23 | -1.25 | 0.71 | -1.22 | 3.71 | 5.98 |
| 49 | ZH161398 | -4.55 | -2.58 | 1.53 | **3.64** | -3.48 | 2.34 | -0.66 | -3.65 | 4.92 | -2.31 | -2.79 | -3.48 | 3.77 | **2.95** |
| 50 | ZH161410 | -3.29 | -2.54 | -0.03 | 5.02 | -3.70 | 4.78 | -1.22 | -3.14 | **3.63** | -1.45 | -2.12 | -2.12 | 4.09 | 4.46 |
| 51 | ZH161478 | -2.10 | -2.69 | 2.36 | 4.95 | -3.07 | 1.14 | -2.22 | -0.85 | 5.95 | -0.31 | -0.95 | -1.91 | 3.01 | 5.61 |
| 52 | ZH161456 | -4.12 | -1.53 | -1.51 | 6.26 | -3.08 | 0.98 | -0.31 | -2.42 | 6.28 | -0.74 | -1.99 | -0.47 | 1.34 | 6.69 |
| 53 | ZH161484 | -4.24 | -2.24 | 0.84 | 4.33 | -3.67 | 3.00 | -1.71 | -3.56 | **3.87** | -2.12 | -2.55 | -1.86 | 0.35 | 5.56 |
| 54 | ZH161463 | -2.00 | -2.89 | -0.53 | 5.91 | -2.86 | 3.17 | -0.32 | -2.55 | 5.27 | -1.06 | -0.76 | -2.14 | 0.87 | 5.76 |
| 55 | ZH15449 | -4.42 | -2.81 | -1.26 | 5.20 | -3.61 | 2.78 | -1.49 | -1.82 | 4.51 | -2.99 | -2.20 | -1.69 | 2.60 | 4.23 |
| 56 | ZH161196 | -3.60 | -1.33 | -0.46 | 5.94 | -4.01 | 1.46 | -1.03 | -2.29 | 5.15 | -1.93 | -2.17 | -1.37 | 2.14 | 5.09 |
| 57 | ZH161194 | -2.26 | -1.50 | -0.58 | 6.54 | -2.69 | 1.11 | -0.34 | -0.44 | 7.03 | -1.07 | -1.12 | -0.92 | 0.61 | 6.59 |
| 58 | ZH161064 | -3.77 | -4.13 | 0.69 | **3.45** | -3.80 | 3.02 | 0.44 | -1.96 | 5.45 | -3.01 | -0.48 | -2.07 | 2.81 | **4.14** |
| 59 | ZH161068 | -4.34 | -5.01 | -0.51 | **3.76** | -3.80 | 2.56 | -1.15 | -1.26 | 4.92 | -4.08 | -0.56 | -1.55 | 3.27 | 4.26 |
| 60 | ZH161129 | -3.51 | -4.23 | 1.12 | **3.34** | -4.84 | 2.62 | -0.09 | -1.10 | 5.16 | -2.82 | -0.58 | -2.18 | 2.96 | **4.05** |
| 61 | ZH161077 | -3.25 | -2.82 | -1.34 | 5.73 | -4.90 | 2.83 | -0.11 | -3.13 | 4.40 | -2.56 | -0.77 | -1.88 | 3.05 | 4.32 |
| 62 | ZH161066 | -0.46 | -3.15 | 0.94 | 6.38 | -3.96 | 3.09 | -0.43 | -1.25 | 5.01 | -1.85 | -0.50 | -1.36 | 1.56 | 5.59 |
| 63 | ZH161137 | -3.82 | -3.60 | -1.46 | 5.21 | -2.11 | 3.90 | -0.93 | -1.01 | 5.78 | -3.05 | -1.75 | 0.07 | 1.46 | 6.19 |
| 64 | ZH161078 | -3.67 | -3.49 | 1.00 | **3.67** | -3.87 | 2.58 | -1.33 | -2.08 | 4.44 | -2.98 | -1.48 | -2.38 | 1.55 | **4.16** |
| 65 | ZH161114 | -2.93 | -1.19 | 0.46 | 5.92 | -1.13 | 2.24 | -0.12 | -3.55 | 6.89 | -1.13 | -1.89 | -1.23 | 2.12 | 5.63 |
| 66 | ZH161043 | -2.91 | -3.18 | -0.11 | 4.93 | -2.63 | 3.33 | -0.67 | -1.34 | 5.54 | -3.15 | 0.26 | -1.79 | 1.12 | 5.32 |
| 67 | ZH161184 | -2.27 | -4.38 | -0.58 | 5.20 | -3.60 | 3.10 | -0.15 | -0.68 | 5.65 | -1.37 | 0.07 | -1.55 | 3.62 | 5.41 |
| 68 | ZH161050 | -3.08 | -1.17 | 1.12 | 5.63 | -2.19 | 1.67 | -1.61 | 0.04 | 6.79 | -2.39 | -2.00 | -1.19 | 0.62 | 5.74 |
| 69 | ZH161207 | -2.90 | -3.58 | 0.01 | 4.67 | -2.33 | 2.58 | 0.33 | -1.71 | 6.44 | -2.77 | -0.61 | -1.17 | 1.47 | 5.40 |
| 70 | ZH161089 | -2.18 | -3.14 | -0.53 | 5.67 | -3.06 | 3.03 | -0.89 | -0.87 | 5.46 | -2.80 | -0.49 | -0.66 | 1.34 | 5.89 |
| 71 | CAH 153 | -4.07 | -3.26 | 0.28 | 3.98 | -4.23 | 2.29 | 0.61 | -1.87 | 5.72 | -2.11 | -2.66 | -1.90 | 2.27 | 4.56 |
| 72 | CAH 1511 | -3.01 | -2.41 | 0.56 | 4.96 | -5.35 | 2.48 | -1.17 | -1.31 | 4.31 | -1.62 | -2.44 | -2.04 | 3.90 | 4.41 |
| 73 | MG 900 | -3.99 | -3.74 | -0.29 | 4.16 | -3.35 | 2.54 | -2.43 | -3.06 | 4.12 | -2.85 | -2.58 | -1.05 | 3.23 | 4.75 |
| 74 | P3502 | -4.20 | -3.33 | -0.73 | 4.60 | -4.29 | 3.14 | -1.82 | -3.78 | **3.33** | -2.39 | -2.84 | -1.52 | 2.63 | 4.66 |
| 75 | Hytech5106 | -3.46 | -3.24 | 0.43 | 4.24 | -3.74 | 2.56 | -0.31 | -2.22 | 5.07 | -2.12 | -1.60 | -2.14 | 3.31 | **4.10** |
| **ID1** | | -6.03 | -5.66 | 2.78 | - | -6.40 | 4.98 | -3.61 | -3.61 | - | -4.40 | -2.10 | -5.46 | 3.90 | - |

**¶**bold values indicating selected hybrids under each moisture condition by assuming ⁓15% selection pressure


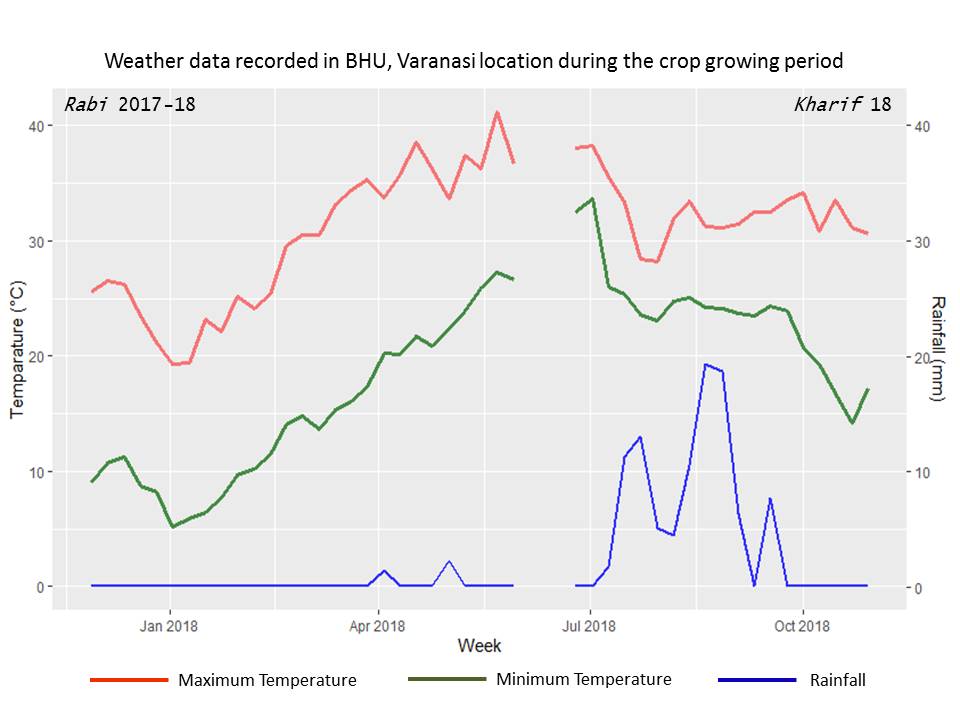


**Supplemental Figure 1a: Weather data on weekly basis recorded at Varanasi location during the crop growing period winter 2017-18 and summer-rainy 2018 seasons**


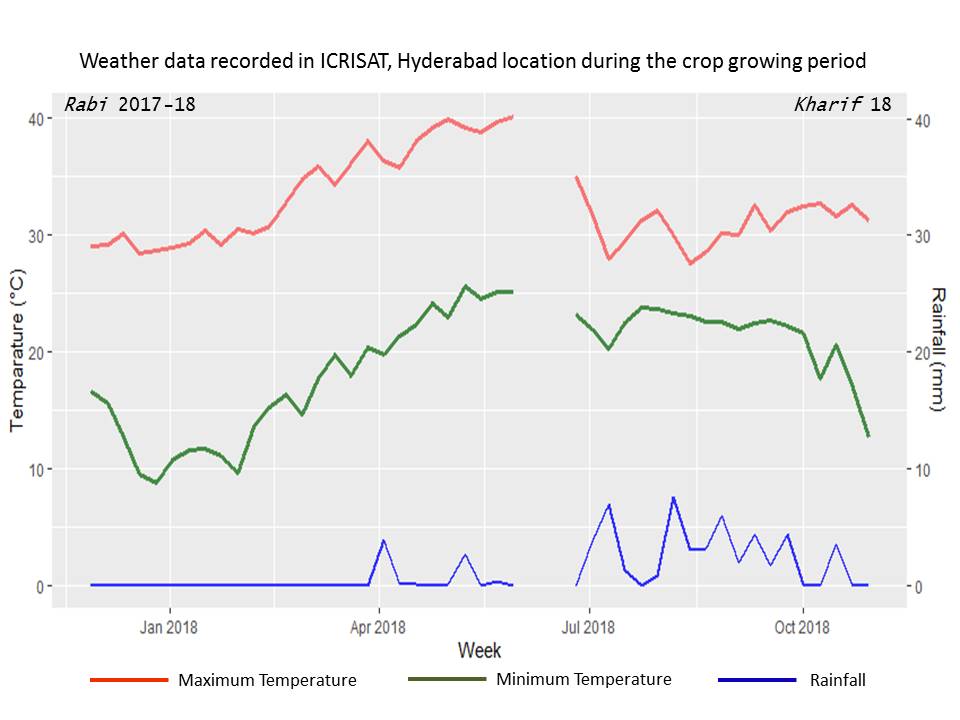


**Supplemental Figure 1b: Weather data on weekly basis recorded at Hyderabad location during the crop growing period winter 2017-18 and summer-rainy 2018 seasons**
